# Pharmacokinetics–pharmacodynamics issues relevant for the clinical use of beta-lactam antibiotics in critically ill patients

**DOI:** 10.1186/s13054-018-2155-1

**Published:** 2018-09-24

**Authors:** Rui Pedro Veiga, José-Artur Paiva

**Affiliations:** 1 Centro Hospitalar São João, EPE – Intensive Care Department, Porto, Portugal; 2 Faculty of Medicine – University of Porto, Porto, Portugal; 3 Grupo Infeção e Sepsis, Porto, Portugal

**Keywords:** Critical care or intensive care or critically ill, Sepsis or septic shock, Antibiotics, Pharmacokinetics, Pharmacodynamics

## Abstract

Antimicrobials are among the most important and commonly prescribed drugs in the management of critically ill patients and beta-lactams are the most common antibiotic class used. Critically ill patient’s pathophysiological factors lead to altered pharmacokinetics and pharmacodynamics of beta-lactams.

A comprehensive bibliographic search in PubMed database of all English language articles published from January 2000 to December 2017 was performed, allowing the selection of articles addressing the pharmacokinetics or pharmacodynamics of beta-lactam antibiotics in critically ill patients.

In critically ill patients, several factors may increase volume of distribution and enhance renal clearance, inducing high intra- and inter-patient variability in beta-lactam concentration and promoting the risk of antibiotic underdosing. The duration of infusion of beta-lactams has been shown to influence the fT > minimal inhibitory concentration and an improved beta-lactam pharmacodynamics profile may be obtained by longer exposure with more frequent dosing, extended infusions, or continuous infusions.

The use of extracorporeal support techniques in the critically ill may further contribute to this problem and we recommend not reducing standard antibiotic dosage since no drug accumulation was found in the available literature and to maintain continuous or prolonged infusion, especially for the treatment of infections caused by multidrug-resistant bacteria.

Prediction of outcome based on concentrations in plasma results in overestimation of antimicrobial activity at the site of infection, namely in cerebrospinal fluid and the lung. Therefore, although no studies have assessed clinical outcome, we recommend using higher than standard dosing, preferably with continuous or prolonged infusions, especially when treating less susceptible bacterial strains at these sites, as the pharmacodynamics profile may improve with no apparent increase in toxicity.

A therapeutic drug monitoring-guided approach could be particularly useful in critically ill patients in whom achieving target concentrations is more difficult, such as obese patients, immunocompromised patients, those infected by highly resistant bacterial strains, patients with augmented renal clearance, and those undergoing extracorporeal support techniques.

## Background

Antimicrobials are among the most important and commonly prescribed drugs in the management of critically ill patients and beta-lactams are the most common antibiotic class used because of their broad spectrum of activity and high tolerability [1, 2].

Early and appropriate antibiotic administration improves clinical outcome of septic patients [3–7]. In the presence of septic shock, besides conflicting results [8, 9], each hour delay is associated with a measurable increase in mortality and other negative endpoints (e.g., length of stay in ICU, acute kidney injury, acute lung injury, and global organ injury assessed by the Sepsis-Related Organ Assessment score) [10, 11].

Choosing the appropriate antimicrobial for the bacterial activity spectrum is crucial but the correct dosage regimen (both dose and frequency) is, at least, of the same importance for successful clinical cure and microbiological eradication [11].

Unlike organotropic drugs, where it is easy to titrate dose to achieve a clinical response, antibiotics may take 24–72 h to present signs of resolution of infection, making it difficult to determine the most appropriate dosage [1, 2].

**Fig. 1:**
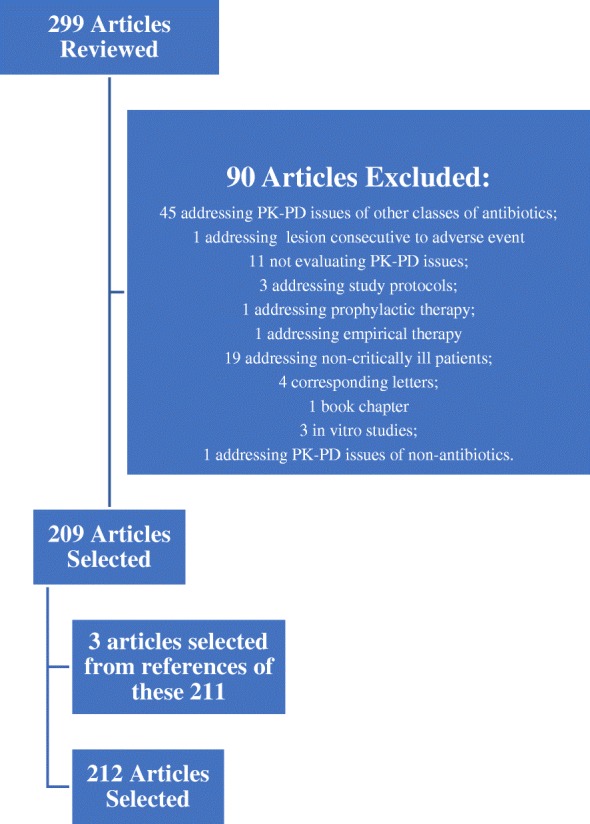
Articles reviewed, included, and excluded

We conducted a comprehensive bibliographic search in the PubMed database of all English language articles published from January 2000 to December 2017, using the following keywords: critical care or intensive care or critically ill and sepsis or septic shock and antibiotics and pharmacokinetics or pharmacodynamics. Articles not addressing beta-lactam pharmacokinetics (PK) or pharmacodynamics (PD) in critically ill patients were excluded. A small number of articles derived from references in the articles selected were also reviewed. In the end, 214 studies were included in our review (Fig. 1).

## Beta-lactam PD characteristics

Knowledge of the antimicrobial PD characteristics (inhibition of growth, rate and extent of bactericidal action, and post-antibiotic effect (PAE)) provides a more rational basis for determination of optimal dosing regimens in terms of the dose and the dosing interval.

The antimicrobial activity of drugs is usually assessed by determination of the minimum inhibitory concentration (MIC) and the minimum bactericidal concentration (MBC) of the drug under specific conditions in vitro. These in vitro conditions are very different from those expected at the site of infection, where the milieu is frequently acidic and anaerobic, and tissue protein may bind a variable amount of the drug. Additionally, these parameters do not provide information on the time course of the antimicrobial effect—the fluctuating levels that are present in a patient treated with the drug—and are measured against a standard bacterial inoculum (about 10 colony-forming units (CFU) per millilitre [5]) that does not necessarily correspond to bacterial densities at site of infection (10 CFU per gram of tissue or pus [8–10]). For bactericidal drugs, the MBC is generally not more than fourfold higher than the MIC. The size of the residual bacterial population at the end of each dosing interval, and ultimately the efficacy of the antimicrobial regimen, depends on the interplay of a variety of bacterial, drug, and host factors that include the size of the initial bacterial population, the potency (MIC and MBC) and PK characteristics of the antimicrobial agent, the rate and extent of any bactericidal effect, the presence of a post-antibiotic effect (PAE), the rate of re-growth of persistent organisms, and the state of host defences [13].

Three PD indices describe optimal killing associated with antibiotics: fT > MIC, which is the amount of time that the unbound drug concentration remains above the MIC of the infecting organism; C_max_/MIC, which is the ratio between the maximum concentration of the drug and the MIC of the infecting organism; and AUC_0–24_/MIC, which is the ratio between total area under the concentration–time curve (AUC) over 24 h and the MIC of the infecting organism.

Beta-lactams are time-dependent antimicrobials whose efficacy is mainly related to fT > MIC [1, 2, 12–14]. Increasing drug concentrations much above the MBC does not enhance bacterial killing and the bactericidal action of these drugs is relatively slow. When drug levels at the site of infection fall below the MIC, the relatively large residual population can resume growth quickly because most beta-lactams either have no or only a short PAE [12]. McKinnon et al. compared the PD of cefepime and ceftazidime and observed that patients with fT > MIC of 100% had significantly greater rates of clinical cure and bacteriological eradication than patients with fT > MIC of < 100% [15].

It is suggested that 50% fT > MIC of the dosage interval is needed to ensure standard efficacy with these antimicrobials, whereas 100% fT > MIC of the dosage interval should be ensured for optimal exposure in immunocompromised patients. A further improvement in efficacy is observed when antibiotic concentrations are four to five times greater than MIC [2, 12, 13]. The percentage of time above MIC that correlates with efficacy varies among different beta-lactam groups, being greater for cephalosporins and aztreonam than for penicillins, and greater for penicillins than for carbapenems. Also, variations occur among different bacterial species, being less for staphylococci, for which beta-lactams have a PAE, than for streptococci and Gram-negative bacilli, for which beta-lactams do not have a PAE [2, 13].

## Beta-lactam PK issues in the critically ill

Discovered antibiotics are evaluated in vitro and tested in animals, initially for toxicity and subsequently for efficacy. The antibiotic dose and frequency are based on these in vitro or animal in vivo PK/PD studies. These dosing regimens are then tested on healthy human volunteers for tolerability, with clinical efficacy studies undertaken in non-critically ill patients. After the launch of the drug onto the general market, the same dosing regimen is used in critically ill patients; however, this is likely to lead to suboptimal outcomes in the ICU [5], especially with more resistant bacterial strains [16] and in the immunocompromised population [17].

Beta-lactams are hydrophilic drugs and so their volume of distribution (Vd) is low and similar to that of extracellular water. Variations in the extracellular fluid content and/or in renal or liver function may be considered the most relevant and frequent pathophysiological mechanisms possibly affecting drug disposition in critically ill patients. Other factors may contribute to altered antibiotic concentrations: an interesting case-report by Taccone et al. [18] related the case of an obese septic patient with *Pseudomonas aeruginosa* pneumonia treated with meropenem. The PD target (*t* > 4 × MIC > 40% of the dosing interval) was only achievable by dosing 3 g q6h at 3 h extended infusion and was associated with clinical improvement.

Compared with healthy volunteers and non-critically ill patients, in critically ill patients capillary leakage and edema, fluid therapy, pleural effusion, ascites, indwelling post-surgical drainage, and hypoalbuminemia may increase Vd and cause antibiotic dilution in plasma and extracellular fluids. Some pathophysiological factors may also enhance (trauma, burns, the hyperdynamic condition of the early phase of sepsis, the use of hemodynamically active drugs) or reduce (renal failure, muscular wastage, bedridden patients) renal clearance and consequently may alter plasma and extracellular antibiotic concentrations (with implications on time over MIC), induce high intra- and inter-patient variability, and promote the risk of antibiotic underdosing [1, 2, 12, 14, 19–35]. Extracorporeal support techniques also contribute to antibiotic concentration variability [36].

PK studies on ICU septic patients reported, overall, increased Vd with significant daily concentration fluctuations between and within patients [5, 36–41]. Clearance of drugs is also affected and usually related to creatinine clearance [1, 42–44]. A single-center study of 17 ICU patients with ventilator-associated pneumonia (VAP) described the PK profile of ertapenem and concluded that, because of its highly protein-bound profile, hypoalbuminemia resulted in a higher protein-unbound fraction with consequences for drug distribution and elimination [38]. Ulldemolins et al. [39] found the same while studying the PK profile of flucloxacillin. Ramon-Lopez et al. [45] described high PK variations (between and within patients) for meropenem in 12 burn ICU patients that were mostly related to age, body weight, and serum albumin. Carlier et al. [37] investigated the adequacy of piperacilin/tazobactam dosing and its trough variability during an entire 7-day antibiotic course in 11 ICU patients with pneumonia and normal renal function. Six of them failed to achieve the PK/PD target of 100% fT > MIC at least once during the treatment course and considerable antibiotic concentration variability was found within and between patients. The *DALI study*, a large multicenter prospective study evaluated 248 ICU patients treated for infection with beta-lactams and found large variations on beta-lactam blood concentrations. The achievement of the PK/PD targets was highly inconsistent, with one fifth of the patients not achieving their most conservative PK/PD target of 50% fT > MIC and better outcomes were described with higher drug exposure, at least for less severely ill patients [5].

Septic patients with acute renal failure may have suboptimal antibiotic concentrations in the first days of therapy when the recommended dosing adjustment for renal failure is used [46]. Taconne et al. [40] studied the PK profiles of four beta-lactams (ceftazidime, cefepime, piperacilin/tazobactam, and meropenem) over the first 24 h of treatment in 80 septic ICU patients. They concluded that, besides high intra- and inter-patient PK variability, standard first doses of broad-spectrum β-lactams provided inadequate levels to achieve target serum concentrations for extended periods of time.

Augmented renal clearance has probably more impact than altered Vd on the PK of beta-lactams [25, 27, 47–54]. Roberts et al. [23] described the PK of cefazolin in plasma and interstitial fluid of subcutaneous tissue in post-trauma critically ill patients and demonstrated that increasing creatinine clearance (ClCr) or decreasing serum albumin concentrations will reduce the likelihood of achieving optimal cefazolin exposure in subcutaneous interstitial tissue. In the presence of augmented renal clearance (ClCr > 130 mL/min), a much higher dose of cefazolin is required to obtain similar relative drug exposures [23]. Conil et al. [43] found that higher ClCr values (> 50 mL/min) did not provide trough concentrations of piperacilin (4 g three times a day) sufficient enough to attain the MIC for many pathogens in many of the patients studied.

Hypoalbuminemia has also been associated with altered PK. Wong et al. [55] described a linear correlation between the percentage protein binding of flucloxacillin and the plasma albumin concentration, though this was not true for ceftriaxone. Also, plasma albumin concentrations and in vitro binding data from healthy volunteers should not be used to predict unbound concentrations of ceftriaxone in ICU patients [56].

## Use of extracorporeal support techniques in critical care

Acute kidney injury (AKI) occurs in 50 to 65% of critically ill patients and in approximately two-thirds of patients within the first 24 h after admission to the intensive care unit (ICU) [57]. Critically ill patients are usually supported with one of the forms of continuous renal replacement therapy (CRRT)—continuous venous-venous hemofiltration, hemodiafiltration, hemodialysis (CVVHF, CVVHDF, CVVHD, respectively)—or with sustained low-efficiency dialysis (SLED). Molecules are transported across the filter membrane by the mechanism of convection (driven by the pressure gradient—CVVHF), diffusion (driven by the concentration gradient—CVVHD, SLED), or both (CVVHDF).

Employing CRRT complicates antibiotic dosing to a significantly higher extent than standard hemodialysis due to the high number of variables, including Vd, flow of the dialysis fluid, replacement fluid infusion site (pre- or post-dilution mode), type and surface of the used membrane, and the difference between delivered and prescribed RRT dose.

Vd in AKI may be significantly different from published population estimates derived from healthy subjects. Besides the decreased plasma protein concentrations in acutely ill patients, uremic solutes, such as hippurate and indoxyl sulfate, alter drug binding to albumin in chronic renal failure and might do so in acute renal failure, although this has not been tested. The free fraction of many drugs is increased in renal failure, even though the Vd for total drug may increase due to movement of unbound drug into interstitial or total body water [57–59].

Overall, a tendency for antibiotic underdosing in critically ill patients on CRRT or SLED likely exists. The mode and dose of CRRT vary quite widely from center to center and from report to report, making it very difficult to create generally applicable beta-lactam dosing guidelines for critically ill patients under CRRT. Additionally, antibiotic concentrations may vary depending on the degree of extraction and residual renal function, which is variable, difficult to assess, and rarely considered despite its relevant contribution to antibiotic clearance in patients undergoing CRRT (Tables 1 and 2) [60–96].

**Table 1:** PK/PD studies of beta-lactams in patients undergoing CRRT

Study	Endpoints	Antibiotic	Design and type of CRRT	Results	Conclusions
Fish et al. [67]	To more fully characterize PK disposition of imipenem in critically ill adult patients during currently used CVVH or CVVHDF regimens	Imipenem-cilastatin	Prospective open-label study of imipenem-cilastatin administered as the combination product in a fixed 1:1 ratio Adult ICU patients with CVVH (*n* = 6 patients) or CVVHDF (*n* = 6 patients) Imipenem administered at 0.5 g every 8 to 12 h (total daily doses of 1 to 1.5 g/day) by intravenous infusion over 30 min Pre- and post-membrane plasma and corresponding ultrafiltrate or dialysate samples were collected at 1, 2, 4, and 8 or 12 h (depending on dosing interval) after completion of the drug infusion	Patients on CVVHDF had significantly higher CLs compared to patients on CVVH (*P* = 0.01), but this difference was not significant when normalized for total body weight (*P* = 0.477) The observed t1/2 was overall similar between both patient groups (*P* = 0.860) No significant differzences were found in Vd, S, and Sa, or ultrafiltration rates Mean Cmax, Cmin, and AUC0–24 values in patients receiving 0.5 g bid during CVVH versus CVVHDF were 17.5 g/mL vs 14.1 g/mL, 1.1 g/mL vs 1.0 g/mL, and 129.5 g·h/mL vs 110.3 g·h/mL, respectively, and in patients receiving 0.5 g tid they were 18.5 g/mL vs 17.1 g/mL, 1.9 g/mL vs 1.1 g/mL, and 183.3 g·h/mL vs 140.6 g·h/mL, respectively Doses of 0.5 g bid and 0.5 g tid generally provided T > MIC of at least 40 to 50% and 50 to 60%, respectively (MICs of < 2 g/mL) Doses of 0.5 g every 6 h (2.0 g/day) were modeled based on the PK parameters observed in this study and the T > MIC calculated: at 2.0 g/day, organisms with a MIC of < 4 g/mL had a T > MIC of at least 50 to 80%. And for those with MIC < 8 g/mL, T > MIC ranged from 34 to 62%	CVVH and CVVHDF contribute to imipenem clearance to a greater degree than previously reported. Imipenem doses of 1.0 g/day appear to achieve concentrations adequate to treat most common Gram-negative pathogens (MIC up to 2 g/mL) but doses of 2.0 g/day or more may be required to adequately treat and prevent resistance in pathogens with higher MICs (MIC > 4 to 8 g/mL)
Malone et al. [68]	To more fully characterize the PK disposition of cefepime in critically ill adult ICU patients during CVVH or CVVHDF	Cefepime	Prospective, observational study 12 adult ICU patients receiving CRRT for severe renal failure (5 patients on CVVH, 7 patients on CVVHDF) Cefepime regimens included either 1 g or 2 g doses administered intravenously every 12 or 24 h (total daily doses of 1 to 4 g/day) Sampling was performed as soon as possible after initiation of the CRRT and drug therapy. Pre- and post-membrane venous blood samples were obtained 1, 2, 4, and 8 h after the completion of the drug infusion and just before administration of the next dose	Drug clearance during CRRT (CL CRRT) and %CLS were significantly higher (*P* = 0.002 and 0.018, respectively), and t1/2 was significantly lower (*P* = 0.005) among patients receiving CVVHDF than in patients receiving CVVH. The mean cefepime S during CVVH and Sa during CVVHDF were estimated at 0.86 ± 0.04 and 0.78 ± 0.10, respectively, indicating that cefepime is extensively cleared across the CRRT membrane Approximately 40 and 59% of cefepime CLs was attributed to membrane clearance during CVVH and CVVHDF, respectively, indicating that the clearance of cefepime was substantially enhanced during both CRRT techniques Values for cefepime Vd were also statistically different (*P* = 0.03) between CVVH and CVVHDF groups, but changes in Vd were only weakly correlated with changes in t1/2 (Spearman’s rank correlation coefficient of 0.641; *P* = 0.133) All pathogens isolated from study patients had cefepime MICs of < 4 g/mL, and doses as low as 1 g/day would predictably provide adequate treatment during either CVVH or CVVHDF Cefepime doses of 2 g/day would be expected to achieve favorable concentrations in serum against susceptible pathogens (MIC < 8 g/mL) with T > MIC greater than 50%. Cefepime (2 g/day) during either CVVH or CVVHDF would also be predicted to achieve favorable T > MIC > 80% against pathogens with intermediate susceptibility (MIC < 16 g/mL)	It appears that CVVHDF is more efficient than CVVH in eliminating cefepime. However, the present study included too few subjects and too much variability was observed within the data to demonstrate this conclusively. Cefepime regimens of 0.25 to 1.0 g/day as recommended by the manufacturer for anuric patients or those receiving conventional hemodialysis would likely be subtherapeutic against all but the most highly susceptible pathogens when administered to patients receiving CRRT The authors recommend cefepime doses of 2 g daily under most circumstances in critically ill patients receiving CRRT. However, considering more resistant strains, doses of 4 g/day should be considered for empirical therapy in life-threatening nosocomial infections, while awaiting results of culture and susceptibility testing
Mueller et al. [69]	To determine the PK of piperacillin-tazobactam in critically ill patients with acute anuric renal failure treated by CVVH	Piperacilin-tazobactam	Prospective, observational study 8 adult ICU patients on CVVHD Doses and dosing schedules were chosen empirically by the attending physicians and were administered intravenously over 15 min Pre-dialyzer blood samples and dialyzer-outlet dialysate samples were taken before drug administration, at 10 and 30 min after infusion, and at 1, 2, 4, 6, 8, 12, 20, 22, and 24 h after infusion	CVVHD clearance of piperacillin was 37% (median, with a range of 13 to 100%) and the CVVHD clearance of tazobactam was 38% (median, with a range of 32 to 92%) of CLtotal Vd was 0.31 ± 0.07 and 0.24 ± 0.09 for piperacilin and tazobactam, respectively t1/2 was 4.3 ± 1.2 and 5.6 ± 1.3 for piperacilin and tazobactam, respectively Simulations of 4 g of piperacillin and 0.5 g of tazobactam administered every 12 h and 2 g of piperacillin and 0.25 g of tazobactam administered every 8 h resulted in times above MIC of > 50% for piperacillin with susceptible (MIC of piperacillin 16 mg/liter; time above MIC, 48 to 100%) and intermediate susceptible (MIC 32 mg/liter; time above MIC, 17 to 100%) pathogens in seven of eight patients	A relevant contribution of CVVHD to the overall elimination of both drugs has to be taken into account The estimated Vd values are greater than those of healthy subjects and t1/2 of both drugs were fourfold greater than those of healthy subjects and twofold greater than those with a creatinine clearance < 20 mL/min/1.73 m^2^ Patients with residual renal function and patients that receive CRRT with higher dialysate flow rates or higher additional hemofiltrate flow rates might have higher CL of piperacillin-tazobactam, resulting in higher dosage needs
Roberts et al. [70]	To assess the variability of antibiotic trough concentrations, the influence of effluent flow rates on such concentrations, and the incidence of suboptimal antibiotic dosage	Meropenem and piperacilin-tazobactam	Prospective, observational, multicenter study, conducted within the multicenter RENAL study. It assessed the effect of post-dilutional higher intensity (40 mL/kg/h effluent rate) or lower intensity (25 mL/kg/h effluent rate) CRRT. Patients were randomized to receive either higher or lower intensity effluent flow rate 24 adult ICU patients with AKI on CVVH. 17 patients were on meropenem and 7 patients on piperacilin-tazobactam Antibiotic dosing was at the discretion of the treating physician: meropenem 0.5 g every 12 h to 1 g every 8 h; piperacilin 4 g every 12 h to 4 g every 6 h Blood samples were obtained on more than one occasion on different days and before administration of the antibiotic to determine the trough concentration	There was marked variability in trough concentrations for all antibiotics: 6.7-fold for meropenem; 3.8-fold for piperacillin; 10.5-fold for tazobactam When investigating trough concentration variability according to effluent flow rate, no statistically significant differences were found using univariate analysis Meropenem trough concentrations were 10.1 ± 8.7 and 15.0 ± 6.4 for low and high effluent flow rates, respectively Piperacilin trough concentrations were 83.6 ± 63.8 and 204.0 ± 105.0 for low and high effluent flow rates, respectively 100% T > MIC was achieved with meropenem (for MIC 2 mg/L) and piperacilin-tazobactam (for MIC 16 mg/L) 76% T > MIC was achieved for meropenem (for MIC 8 mg/L) 86% T > MIC was achieved for piperacilin-tazobactam (for MIC 64 mg/L)	It appears that CRRT effluent flow rates cannot be used independently to guide dose adjustment Trough concentrations failed to achieve the higher therapeutic target in 24% of patients receiving meropenem and 14% of patients receiving piperacillin, which is of concern Dose-adjusting to achieve a target concentration that exceeds the appropriate MIC but is less than potentially toxic concentrations seems desirable but cannot be reliably achieved with empirical dosing
Banyai et al. [71]	To study the PK of cefpirome in critically ill patients with acute kidney failure treated by CVVH and to develop an optimal dosing regimen in patients with CVVH	Cefpirome	Prospective, observational study 8 adult ICU anuric patients with acute kidney failure on CVVH All patients received a dosage of 2 g cefpirome over a period of 30 min, every 8 h after starting the hemofiltration Blood samples were collected from the arterial and venous line of the extracorporeal circuit immediately at baseline and at 60, 120, 180, 240, 300, 360, and 420 min after the start of the first infusion. Additional blood samples were collected immediately before the end and 30 min after the end of each infusion, up to a total study period of 48 h Ultrafiltration samples were collected from the outlet of the ultrafiltrate compartment of the hemofilter at corresponding times	C_max_ 14.8 ± 3.2 μg/mL (10.8 to 19.7) C_min_ 3.1 ± 0.8 μg/mL Post hemofiltration to pre-hemofiltration ratio of 0.23 ± 0.10 Total drug removal of 78.0% ± 8.8% Elimination t1/2 was 2.36 ± 0.59 h (1.6 to 3.2 h). The calculated Vd was 118 ± 36 L Total body clearance and hemofiltration clearance were 589.1 ± 164.5 mL/min and 43.3 ± 7.8 mL/min, respectively The calculated AUC was 60.4 ± 16.0 mg/L·h	Highest levels of cefpirome were significantly lower compared with values observed in healthy volunteers and in patients on hemodialysis Plasma cefpirome concentrations remained above 4 μ g/mL for 62% and above 8 μ g/mL for 25% of the dosing interval, respectively In patients infected with an intermediate susceptible *Pseudomonas aeruginosa* (MIC90 ~ 8 mg/L), no sufficient antimicrobial efficacy can be expected Comparable low trough levels of 3.1 ± 0.8 μ g/mL suggest a dosage recommendation of 2 g cefpirome every 8 h
Eyler et al. [72]	To determine the PK of ertapenem in critically ill adults receiving CVVHD or CVVHDF	Ertapenem	Prospective, open-label, first-dose PK study 8 adult ICU patients with suspected or confirmed Gram-negative infections receiving CVVHD (4 patients) or CVVHDF (4 patients) 1 g ertapenem was administered as a half-hour intravenous infusion Blood samples were collected from the CVVHD circuit at the sampling port, just before the hemodialysis filter at time zero (baseline), 30 min (end of infusion), and 1, 2, 4, 8, 12, 18, and 24 h after the start of the ertapenem infusion. At the same time points, effluent was also collected from the effluent port of the CVVHD/F circuit	CLS, unbound 48 mL/min VC, unbound 32 l VP, unbound 21 l CLdial, unbound 36 mL/min PTA (40%T > MIC) and fraction of the dosage interval spent above the MIC for different regimens were For MIC of 0.5 mg/L 500 mg q12h, 1.0 and 0.999 500 mg q24h, 1.0 and 0.999 750 mg q24h, 1.0 and 0.999 1000 mg q24h, 1.0 and 0.999 For MIC of 1 mg/L 500 mg q12h, 1.0 and 0.999 500 mg q24h, 0.99 and 0.916 750 mg q24h, 1.0 and 0.999 1000 mg q24h, 1.0 and 0.999 For MIC of 2 mg/L 500 mg q12h, 1.0 and 0.992 500 mg q24h, 0.962 and 0.563 750 mg q24h, 0.999 and 0.750 1000 mg q24h, 0.999 and 0.917	The unbound fraction (20 to 40%) was markedly increased compared to those reported for healthy volunteers (5 to 15%) At the effluent rates, ertapenem was cleared to a substantial degree During the study sampling period, the dose of 1 g every 24 h produced unbound ertapenem concentrations that remained above 2μg/mL for an average of 90% of the dosing interval, achieving the PD targets in all eight patients Monte Carlo simulations revealed that 99.9% of simulated subjects would achieve unbound ertapenem concentrations above 2μg/mL for at least 40% of the interval, with concentrations remaining above 2μg/mL for a median of 92% (range, 33 to 100%) of the dosing interval Particularly in patients where organisms with high MICs are suspected, it may be necessary to use doses > 500 mg q24h. Concentrations remained above 2μg/mL for an interquartile range of only 50 to 67% of the dosing interval
Vossen et al. [73]	To characterize the PK profile of 1000 mg doripenem q8h for critically ill patients receiving CRRT	Doripenem	Prospective, open-label, observational study 13 adult ICU patients under CRRT (5 on CVVH, 5 on CVVHD, 3 on CVVHDF) All patients received 1000 mg every 8 h at a 30-min infusion Blood and dialysate samples were drawn from the arterial (input), venous (output), and effluent dialysate ports of the dialysis machine before the first administration of doripenem and at 0.5, 1, 2, 3.5, 7, 8, 9, 16, 17, 24, 24.5, 25, 26, 27.5, 31, 32, 33, 40, 41, and 48 h following the start of the first infusion	All patients: AUC0–8 (mg·h/L) 78.58 ± 10.32; Cltot (L/h) 8.07 ± 1.77; Clpre-post filter (mL/min) 36.06 ± 14.27; Sc 0.150 ± 0.053; ClSc (mL/min) 5.20 ± 1.95; Vd total 59.26 ± 26.47; t1/2 (h) 5.39 ± 2.84 Patients on CVVH (*n* = 3): AUC0–8 (mg·h/L) 87.15 ± 8.13; Cltot (L/h) 6.53 ± 1.00; Clpre-post filter (mL/min) 63.82 ± 5.83; Sc 0.129 ± 0.033; ClSc (mL/min) 6.45 ± 1.81; Vd total 51.05 ± 10.18; t1/2 (h) 5.72 ± 1.96 Patients on CVVHD (*n* = 5): AUC0–8 (mg·h/liter) 77.59 ± 12.31; Cltot (L/h) 7.48 ± 1.43; Clpre-post filter (mL/min) 34.03 ± 7.22; Sc 0.164 ± 0.067; ClSc (mL/min) 5.63 ± 2.30; Vd total 71.01 ± 34.98; t1/2 (h) 6.80 ± 3.37 Patients on CVVHDF (*n* = 5): AUC0–8 (mg·h/L) 76.15 ± 4.50; Cltot (L/h) 9.29 ± 1.28; Clpre-post filter (mL/min) 26.99 ± 5.34; Sc 0.145 ± 0.035; ClSc (mL/min) 4.68 ± 1.43; Vd total 50.80 ± 6.34; t1/2 (h) 3.87 ± 0.80 The simulations conducted show that the proposed dose level of 1000 mg every 8 h is superior to lower doses for reaching the desired plasma doripenem concentration of 8 mg/L. Although mean trough concentrations in all dosing regimens exceeded 4 mg/L during steady state, the individual outcomes were highly variable At a dose of 500 mg every 8 h, only 39.5% of the simulated patients showed trough concentrations that were constantly above the lower threshold (4 mg/L) during steady state. At a dose of 1000 mg every 8 h, plasma concentrations still fell below 8 mg/L, but not lower than 4 mg/L, for 60.5% of the simulated patients	The mean hemofilter clearance rates observed slightly exceeded those reported in the literature The sieving coefficients observed differed dramatically from those reported previously The prefilter/postfilter clearance values found in our trial are within the range of values reported previously for imipenem and meropenem There was an uncharacteristically low clearance for CVVHDF patients, which may be attributed to the larger membrane size and higher membrane kUF employed for the CVVHF and CVVHD groups. The usual expectation for beta-lactam antimicrobials would be quite the opposite: CVVHDF clearance values should be higher than CVVHD or CVVH clearance values. However, this is true only if the same flow rates and membrane materials are chosen. If the 1-g q8h regimen is used, 39.5% of the patients will reach a trough level of 8 mg/L at the end of each dosing interval. To attain sufficient drug exposure during the first dosing interval the administration of an initial “loading dose” of 20.4 mg/kg of body weight is suggested The broad therapeutic index of beta-lactams favors higher dosing, providing safety margin for more-effective RRT modalities
Carlier et al. [74]	To describe the PK of cefepime in septic shock patients requiring CRRT and to investigate whether PK/PD targets are achieved with current dosing strategies as well as to investigate the potential advantages of alternative dosing regimens	Cefepime	Prospective, observational study 13 adult ICU patients with septic shock and on CRRT (CVVHF or CVVHDF) Patients received 2 g q8h or q12h. The dose was administered as a 30-min intravenous infusion Blood samples were drawn on the day of inclusion and then every second day during CRRT treatment whenever possible. On each sampling day, blood samples were drawn immediately before antibiotic administration (0 h) and then at 1, 2, and 5 h and at 6 or 12 h (depending on the antibiotic regimen) after the start of the infusion	CL (L/h) 4.5 Vd (L) 40.6 PTA at different UFR considering a MIC of 16 mg/L UFR 1000 mL/h 1 g q12h 100%T > MIC 64% and 60%T > MIC 95% 2 g q12h 100%T > MIC 89% and 60%T > MIC 99% 1 g q8h 100%T > MIC 95% and 60%T > MIC 100% 2 g q8h 100%T > MIC 99% and 60%T > MIC 100% 1 g q6h 100%T > MIC 100% and 60%T > MIC 100% UFR 1500 mL/h 1 g q12h 100%T > MIC 31% and 60%T > MIC 80% 2 g q12h 100%T > MIC 82% and 60%T > MIC 98% 1 g q8h 100%T > MIC 87% and 60%T > MIC 99% 2 g q8h 100%T > MIC 96% and 60%T > MIC 100% 1 g q6h 100%T > MIC 97% and 60%T > MIC 100% UFR 2000 mL/h 1 g q12h 100%T > MIC 9% and 60%T > MIC 51% 2 g q12h 100%T > MIC 73% and 60%T > MIC 95% 1 g q8h 100%T > MIC 79% and 60%T > MIC 95% 2 g q8h 100%T > MIC 92% and 60%T > MIC 100% 1 g q6h 100%T > MIC 93% and 60%T > MIC 100%	Antibiotic clearance was proportional to UFR, with important variability between patients both for clearance and Vd A dose of 2 g q8h or 1 g q6h leads to optimal target attainment (100% T > MIC) whilst minimizing the probability of reaching toxic trough concentrations for patients treated with a high UFR (1500–2000 mL/min). However, the optimal dose for patients treated with lower UFRs (≤ 1000 mL/h) when aiming for the high target was 1 g q8h
Seyler et al. [75]	To evaluate whether the recommended doses of broad-spectrum beta -lactams result in appropriate serum concentrations in ICU patients with severe sepsis and septic shock receiving CRRT	Meropenem Piperacilin-tazobactam Cefepime Ceftazidime	Prospective, open-label study 53 adult ICU patients with severe sepsis or septic shock on CRRT (CVVH, *n* = 19 or CVVHDF, *n* = 34) Meropenem 1 g q12h (*n* = 17) Piperacilin-tazobactam 4.5 g q6h (*n* = 16) Cefepime 2 g q12h (*n* = 8) Ceftazidime 2 g q12h (*n* = 12) Each antibiotic dose was administered as a 30-min infusion Serum concentrations of these antibiotics were determined from samples taken before (*t* = 0) and 1, 2, 5, and 6 or 12 h (depending on the bea-lactam regimen) after the administration of each antibiotic Series of measurements were separated into those taken during the early phase (< 48 h from the first dose) of therapy and those taken later (> 48 h)	Meropenem Vd (l/kg) 0.45 (0.20 to 3.03) Cmax (μg/mL) 26 (15 to 67) Cmin (μg/mL) 6 (2 to 11) AUC (mg/h/mL) 134 (61 to 291) CL (mL/min/kg) 1.15 (0.54 to 3.37) t1/2 (h) 4.39 (2.61 to 30.5) Piperacilin-tazobactam Vd (l/kg) 0.44 (0.22 to 1.72) Cmax (μg/mL) 138 (36 to 262) Cmin (μg/mL) 60 (4 to 155) AUC (mg/h/mL) 527 (62 to 1378) CL (mL/min/kg) 1.15 (0.27 to 6.26) t1/2 (h) 4.16 (1.05 to 15.3) Cefepime Vd (l/kg) 0.55 (0.33 to 0.94) Cmax (μg/mL) 43 (28 to 83) Cmin (μg/mL) 11 (3 to 22) AUC (mg/h/mL) 379 (148 to 483) CL (mL/min/kg) 1.04 (0.43 to 2.97) t1/2 (h) 6.17 (3.30 to 22.9) Ceftazidime Vd (l/kg) 0.37 (0.22 to 0.84) Cmax (μg/mL) 78 (54 to 118) Cmin (μg/mL) 24 (5 to 46) AUC (mg/h/mL) 536 (258 to 906) CL (mL/min/kg) 0.52 (0.13 to 1.61) t1/2 (h) 7.74 (2.52 to 33.5) PK/PD target attainment (four times MIC attainment for *Pseudomonas* spp.) Meropenem Day < 48 h (*n* = 7) 71% Day > 48 h (*n* = 15) 87% Piperacilin-tazobactam Day < 48 h (n = 12) 66% Day > 48 h (*n* = 9) 78% Cefepime Day < 48 h (n = 7) 0% Day > 48 h (*n* = 4) 0% Ceftazidime Day < 48 h (n = 8) 38% Day > 48 h (n = 7) 71%	The recommended doses for broad-spectrum beta-lactams are generally insufficient to maintain therapeutic serum concentrations greater than four times the MIC of *P. aeruginosa* Applying results to other MICs, the observed concentrations for all antibiotics were adequate in 90% of patients only for MICs lower than the clinical breakpoint of *Pseudomonas* spp., which correspond to MICs of sensitive *Enterobacteriaceae* In the first 48 h of treatment, 29%, 34%, 100%, and 62% of our patients treated with meropenem, piperacillin-tazobactam, cefepime and ceftazidime, respectively, never reached the PK target. After 48 h of treatment, the drug concentrations obtained were higher (significantly different only for meropenem), but they remained insufficient in many patients At the onset of sepsis in patients receiving CRRT, similar beta-lactam doses to those used in the absence of renal failure should be given during the first 48 h of therapy Dose reduction should be considered thereafter to avoid drug accumulation. Considering the large PK variability, therapeutic drug monitoring of beta-lactams should be performed to optimize antibiotic efficacy
Roberts et al. [76]	To evaluate variability in CL and Vd and to assess the effect of CRRT prescription on extracorporeal and systemic antibiotic CL and Vd in patients treated with CRRT of different intensities	Meropenem Piperacillin-tazobactam	Nested cohort prospective multicenter observational PK study within a randomized controlled trial of CRRT intensity Patients were randomly assigned to receive post-dilutional hemodiafiltration as either a higher (40 mL/kg body weight/h effluent flow rate) or lower (25 mL/kg body weight/h effluent flow rate) intensity rate Sampling occurred each day (1) immediately before antibiotic dosing, (2) after completion of their intravenous infusion, and (3) at 4 h after completion of infusion It occurred at 65 time points for meropenem and 29 time points for piperacillin-tazobactam in 24 patients	Mean hemodiafiltration clearance of meropenem, piperacillin, and tazobactam did not differ significantly between higher vs lower CRRT intensity: 23 (16–29) vs 21 (15–28), *P* = 0.4802; 22 (21–31) vs 24 (17–31), *P* = 0.9091; 37 (34–49) vs 56 (41–66), *P* = 0.0642, respectively Systemic clearance and Vd were Meropenem 38 mL/min (23–95) and 17.5 L Piperacillin 59 mL/min (37–115) and 18.7 L Tazobactam 113 mL/min (45–248) and 49.3 L	The prescribed intensity of CRRT did not adequately predict extracorporeal clearance or Sd, CLs, Vd, or half-life Systemic CL and elimination half-life did not differ according to CRRT dose, and so the CRRT prescription may not be useful for guiding antibiotic prescribing. In many cases, extracorporeal CL accounted for more than 30% of the observed systemic CL for that antibiotic, which is a suggested threshold for adjustment of the dosing regimen Drug monitoring may be the most practical method for ensuring that antibiotic therapeutic targets are achieved in critically ill patients receiving CRRT
Ohchi et al. [77]	To investigate PK characteristics of doripenem in patients receiving high-flow vs conventional flow intensity CVVHDF	Doripenem	Prospective, observational study Adult ICU Two patients with AKI on high-flow CVVHDF. Patients on conventional CVVHDF were described in a previous study Doripenem administrated as 250 mg single dose infusion over 1 h Blood samples were collected at 1 (just prior to the end of antibiotic infusion), 2, 3, 4,7, and 12 h after initiating the infusion High flow CVVHDF parameters: blood flow 100 mL/min; dialysate flow rate 1500 mL/h; filtration flow rate 900 mL/h	Conventional CVVHDF AUC 74.15 ± 15.5 mg.h/L Cls 58 ± 12.7 mL/min Cl dialysis 13.5 ± 1.6 mL/min t1/2 7.9 ± 3.7 h High-flow CVVHDF AUC 35.2 mg.h/L Cls 118 mL/min Cl dialysis 41.9 mL/min t1/2 2.9 h	Extracorporeal clearance increases in proportion with the intensity dialysis rate The daily dose thus must be increased to 1.0–1.5 g, the same dosage used when creatinine clearance is > 50 mL/min
Arzuaga et al. [78]	To study the PK of piperacillin and tazobactam during CRRT in ICU patients with various degrees of renal impairment.	Piperacilin-tazobactam	Prospective, observational study 14 adult ICU patients on CVVHDF, grouped according to severity (CLCR 10 mL/min, 10 < CLCR < 50 mL/min, and CLCR> 50 mL/min) Piperacilin 4 g and tazobactam 0.5 g were administrated every 6 or 8 h, by 20-min intravenous infusion Prefilter blood and ultrafiltrate samples were collected at 0, 0.3, 0.5, 0.75, 1, 3, 6, and 8 h (in case of administration every 8 h) after the administration of the antibiotic. Time 0 was considered just before the beginning of the 20- min infusion	CLCR < 10 mL/min (*n* = 4) CLCR: 8.67 ± 2.31 mL/min UF flow: 27.1 ± 7.8 mL/min Sc: PIP 0.42 ± 0.25; TZ 0.76 ± 0.26 Cmax: PIP 365.6 ± 232.3; TZ 38.4 ± 13.4 mg/L t1/2: PIP 7.8 ± 4.2; TZ 7.9 ± 3.0 h Cls: PIP 50.0 ± 53.0; TZ 50.4 ± 38.3 mL/min Hemofiltration Cl: PIP 11.45 ± 6.5 1; TZ 20.9 ± 12.6 mL/min AUC: PIP 76143 ± 49,748; TZ 23218 ± 27,943 mg.h/L Vd: PIP 21.0 ± 11.7; TZ 18.9 ± 7.1 l 10 < CLCR > 50 mL/min (*n* = 5) CLCR 25.20 ± 7.73 mL/min UF flow: 30.3 ± 4.3 mL/min Sc: PIP 0.38 ± 0.37; TZ 0.73 ± 0.32 Cmax: PIP 244.5 ± 122.1; TZ 31.5 ± 5.1 mg/L t1/2: PIP 4.2 ± 2.3; TZ 4.1 ± 0.9 h Cls: PIP 90.6 ± 29.9; TZ 68.2 ± 26.2 mL/min Hemofiltration Cl: PIP 12.2 ± 13.2; TZ 21.9 ± 9.6 mL/min AUC: PIP 45445 ± 25,525; TZ 23218 ± 27,943 mg.h/L Vd: PIP 26.8 ± 19.8; TZ 21.6 ± 3.0 l. CLCR> 50 mL/min (*n* = 5) CLCR: 82.40 ± 20.03 mL/min UF flow: 20.0 ± 7.5 mL/min Sc: PIP 0.23 ± 0.07; TZ 0.86 ± 0.30 Cmax: PIP 160.6 ± 93.2; TZ 15.7 ± 6.6 mg/L t1/2: PIP 2.6 ± 0.8 TZ 5.0 ± 3.9 h Cls: PIP 265.2 ± 152.2; TZ 180.1 ± 73.9 mL/min Hemofiltration Cl: PIP 4.8 ± 3.3; TZ 19.6 ± 15.3 mL/min AUC: PIP 17,328 ± 11,134; TZ 2098 ± 1030 mg.h/L Vd: PIP 44.9 ± 20.4; TZ 60.3 ± 34.6 l t > MIC90 obtained were 100% for all the pathogens in patients with creatinine clearance < 10 mL/min. In patients with a creatinine clearance between 10 and 50 mL/min, t > MIC90 was 100% for pathogens with MIC90 ≤ 32, but only 50% for microorganisms with an MIC90 of 64. However, in patients with creatinine clearance > 50 mL/min, as piperacillin elimination was faster, t > MIC90 was only 55.5% and 16.6% for pathogens with MIC90 values of 32 and 64, respectively	The contribution of the hemofiltration clearance to the total clearance increased with the degree of renal insufficiency. Correct doses of these drugs should take into account this observation to avoid clinical failures due to underdosing For both drugs, significant differences were documented in the majority of the PK parameters when patients with CLCR > 50 mL/min were compared to patients with CLCR ≤ 10 mL/min The observed sieving coefficient of piperacillin and tazobactam plus the effluent amount gave a relevant extracorporeal clearance only in the severe renal impairment group, with more than 25% of total clearance for both drugs To increase the t > MIC90 index, piperacillin-tazobactam combination every 4 h could be a better dosage regimen in patients presenting CLCR > 50 mL/min
Isla et al. [79]	To describe the PK of meropenem in critically ill patients with different degrees of renal impairment undergoing CVVHF or CVVHD	Meropenem	Prospective, observational study 20 adult ICU patients on CVVHF or CVVHDF Grouped into 3 categories according to the renal function: 7 with severe failure, ClCr less than 10 mL/min (group I); 7 with moderate failure, ClCr 10 to 50 mL/min (group II); and 6 with ClCr greater than 50 mL/min (group III) Blood flow rate 110–220 mL/min; dialysate flow rate 500 or 1000 mL/min; ultrafiltrate 800–2500 mL/h Patients received 500, 1000, or 2000 mg of meropenem intravenously every 6 or 8 h and infused over 20 min Blood was obtained from a prefilter device immediately before dosing, at the end of the infusion, and at 20, 30, and 45 min and 1, 3, and 6 h after the beginning of the infusion. Another sample was collected 8 h after the beginning of the infusion in patients to whom meropenem was administered every 8 h Simultaneously, dialysate-ultrafiltrate samples were taken directly from the dialysate-ultrafiltrate device	No significant differences depending on renal impairment were found in the Sc. No differences were found in the Sc obtained by CVVHF and the Sa obtained by CVVHD; both membranes showed a similar permeability to meropenem Total clearance was significantly higher in group III than in the other two groups. This finding could be attributable to the lower t1/2 (1.51 ± 0.52 h in group III versus 2.73 ± 0.68 h and 3.72 ± 0.82 h in groups II and I, respectively) and to the high Vd observed in those patients (1.31 ± 0.90 L/kg in group III, 0.37 ± 0.10 L/kg in group II, and 0.57 ± 0.29 L/kg in group I) The contribution of CRRT to total clearance diminished in the extent that CLCR increased. Although there were no statistically significant differences between groups I and II, Cl_CRRT_ was significantly lower in group III In group I patients, trough plasma concentrations were above 4 μg/mL, with the exception of the only patient who received 500 mg/8 h. In group II, plasma concentrations were above 2 μg/mL during the entire dose interval, except in the patient to whom 1000 mg/8 h was administered In spite of the higher doses the patients of group III received, 4 of 6 patients showed concentrations below 0.5 μg/mL	Differences in meropenem PK in critically ill patients undergoing CRRT with different degrees of renal impairment have been observed, and they should be taken into account when dosing critically ill patients In those patients with no renal impairment the risk of underdosing and clinical failure is important, and the administration of meropenem 2000 mg every 8 h did not reach plasma levels to ensure adequate T > MIC values against many bacteria
Ulldemolins et al. [80]	To describe the PKs of meropenem in critically ill patients with septic shock and CRRT, to identify the sources of PK variability in these patients, and to perform different dosing simulations to assess their probability of target attainment by MIC, in order to provide empirical dosing recommendations based on clinical characteristics	Meropenem	Prospective, observational, multicentre study 30 adult ICU patients with septic shock and CRRT, either CVVHF (*n* = 4) or CVVHDF (*n* = 26) Patients were prescribed meropenem at 500 mg q12h over 30 min (*n* = 1); 500 mg q8h over 30 min (*n* = 2) or as a 3-h infusion (*n* = 3); 500 mg q6h as a 3-h infusion (*n* = 1); 1000 mg q12h over 30 min (*n* = 6) as a 3-h infusion (*n* = 1) or as a 4-h infusion (*n* = 1); 1000 mg q8h over 30 min (*n* = 8) as a 3-h infusion (*n* = 5) or as a 4 h-infusion (*n* = 1); or 2000 mg q8h over 30 min (*n* = 1) Blood samples were collected at 24 h of CRRT and meropenem therapy. For bolus sampling, 6 samples were collected at 10 min pre-dose; at 0, 15, and 60 min and between 3 and 6 h after the end of the infusion; and just before the next dose. For extended infusion sampling, samples were collected at 10 min pre-dose; 0, 60, and 120 min after the end of the infusion; and just before the next dose CRRT settings: the median intensity on the day of the study was 34.7 mL/kg/h (range, 18.7 to 60.1 mL/kg/h), and the median blood flow was 200 mL/min (range, 130 to 250 mL/min)	The study model failed to identify CRRT intensity to be a significant modifier of meropenem CL, which may lead to the hypothesis that even the lowest CRRT intensities studied may be enough to maximize meropenem clearance and that higher intensities may add little to total meropenem CL There were no differences between CRRT techniques, likely because of the underrepresentation of CVVHF (4 out of 30 patients) in the study population For the attainment of a PD target of 100% of the T > MIC, fixed doses would be required, depending on the MIC of the bacteria, but the infusion time would depend on residual diuresis: oligo-anuric patients would benefit from a 30-min bolus, while a 3-h extended infusion would be more appropriate for those patients with preserved diuresis For the attainment of the classic PD target for carbapenems (40% of the T > MIC), a standard dose of 500 mg q8h as a bolus over 30 min would be sufficient for all cases For the attainment of a more aggressive target, such as a Cmin/MIC ratio of 5, doses of 1000 mg q8h as a 3-h infusion or higher would be required	Population PK model successfully identified residual diuresis to be a modifier of total meropenem CL CRRT intensity did not significantly modify meropenem CL, for which dose adjustments based on intensity seem to be unnecessary Given a certain MIC, simulations showed that meropenem dose titration considering residual diuresis was advantageous for the attainment of 100% of the T > MIC as a PD target. If classic PD targets (40% of the T > MIC) were targeted, a standard dose of 500 mg q8h as a 30-min bolus would be sufficient, regardless of urine output
Bouman et al. [81]	To compare the observed Cl_CVVHF_ (calculated from measured data) and the predicted Cl_CVVHF_ (calculated from the F_UP_) To determine whether dose adjustment according to the predicted CVVH removal provides an estimate as reliable as that according to the observed CVVH removal	Amoxicillin Ceftazidime Flucloxacillin	Prospective, observational study 45 adult ICU oligoanuric patients on CVVHF During the sampling period a single antimicrobial drug was administered to 31 patients, two drugs to 9 patients, and a combination of 3 to 5 Dosages were: amoxicilin 1000 mg q6h; ceftazidime 1000 mg q6-12 h; flucloxacillin 2000 mg q4-6 h Blood flow rate was 150 mL/min and warmed substitution fluids were administered in predilution at a flow rate of 2000 mL/h. If a negative fluid balance was required, the ultrafiltration flow was increased and the substitution flow was constant Blood samples were collected from the afferent (pre-hemofilter) and efferent (post-hemofilter) line of the extracorporeal circuit and from the ultrafiltrate line. Samples were collected at 2, 4, and 6 h for agents given every 6 h; at 2, 4, and 8 h for agents given every 8 h; and at 2, 6, and 10 h for agents given every 12 or 24 h	All the studied agents were easily filtered (SC > 0.7) with the exception of flucloxacillin There was a high interindividual variability in the SC values of the studied drugs, in particular for ceftazidime and to a lesser degree for amoxycillin and flucloxacillin The correlation between observed and predicted clearance was significant (*P* = 0.003) only when all drugs were combined, not for the individual antimicrobial drugs. Despite the nonsignificant correlation, the difference between predicted and observed clearance for all drugs was small, with the exception of ceftazidime	There was no significant correlation between predicted and observed CVVH drug removal. However, for clinical practice, dose adjustment according to the predicted CVVH removal provides a more reliable estimate than that according to the observed CVVH removal Although there is interpatient variability between the observed and predicted Cl_CVVHF_ values for some antibiotics, its effect on dosing strategies is not necessarily clinically relevant: flucloxacillin has an important non-renal elimination route, and therefore the Fr_CVVHF_ value was extremely low and not affected by the wide interindividual variation in observed Cl_CVVHF_. Also, wide therapeutic range, such as with ceftazidime, makes it safe to use the predicted Cl_CVVHF_ for dose adjustment

*AKI* acute kidney injury, *AUC* area under the curve, *ClCr* creatinine clearance, *CLs* systemic clearance, *Cl dialysis* extracorporeal clearance, *Cmax* maximal concentration, *Cmin* minimal concentration, *CRRT* continuous renal replacement therapy, *CVVHF* continuous venous-venous hemofiltration, *CVVHD* continuous venous-venous haemodialysis, *CVVHDF* continuous veno-venous hemodiafiltration, *Fup* unbound fraction of a drug, *MIC* minimal inhibitory concentration, *PTA* probability of target attainment, *S* saturation coefficient, *Sc* sieving coefficient, *t1/2* half-life, *T > MIC* time above MIC, *UFR* ultrafiltration rate, *Vd* volume of distribution

**Table 2:** PK/PD studies of beta-lactams in patients with sustained low-efficiency dialysis or extended daily dialysis

Study	Endpoints	Antibiotic	Design	Results	Conclusions
Kielstein et al. [82]	To evaluate PK of meropenem in critically ill patients with renal failure undergoing EDD	Meropenem	Prospective clinical study Adult ICU patients with anuric acute renal failure being treated with EDD and receiving meropenem (*n* = 10) Meropenem administered as 1 g dose, as an intravenous infusion over a period of 30 mins, 6 h before EDD was started Blood samples were drawn before administration of the drug; at 0.5, 1, 2, 4, and 6 h after its administration; before EDD; during EDD, at time points 2, 4, and 6 h; at the end of EDD; and at 0.5, 1, 3, and 8 h after EDD. Additional blood samples were drawn pre- and post-dialyzer in order to calculate the dialyzer clearance	The average (mean ± SD) dialysis time during the study was 480 ± 6 min, and mean blood and countercurrent dialysate flow was 160 ± 3 mL/min T1/2off was 8.7 h [4.7–30] T1/2on was 3.7 h [2.1–4.7] Vd was 0.72 L/kg [0.35–2.78] CLoff 5.01 L/h [2.44–11.15] CLdial was 2.3 L/h [0.7–3.7] (estimated from the drug amount recovered in the dialysate) and 5.1 L/h [4.3–5.7] (estimated from drug concentrations before and after application of the dialysis membrane)	Meropenem is significantly eliminated by EDD. Compared with PK results in the literature for intermittent dialysis and CRRT, dosing regimens cannot be used for critically ill septic patients with renal failure being treated with EDD EDD eliminates meropenem at least to an extent similar to CVVH. Thus, physicians run the risk of underdosing. A dose of 0.5 to 1.0 g meropenem every 8 h is recommended. The exact dose should be tailored according to weight and severity of illness as well as to the current MIC against the incriminated bacteria. Whenever possible, therapeutic drug monitoring should be performed
Lorenzen et al. [83]	The aims of this study were to evaluate the PK of ampicillin/sulbactam in critically ill patients with AKI undergoing extended dialysis and to establish a dosing recommendation for this treatment method	Ampicilin-sulbactam	Prospective, open-label, observational study 12 adult ICU patients with anuric AKI PK after a single dose of ampicillin/sulbactam (2 g/1 g) over a period of 30 min was obtained in 12 patients. Multiple-dose PK after 4 days of twice-daily ampicillin/sulbactam (2 g/1 g) was obtained in three patients. The average dialysis time was 442 ± 77 min and mean blood and counter current dialysate flow was 162 ± 6 mL/min, resulting in a mean urea reduction ratio of 50.1% ± 2.7%. ED was started 3 h after the end of the ampicillin/sulbactam infusion	Cmax 280.9 ± 174.9 mg/L Tmax 0.5 h AUClast 847.5 ± 499.5 mg.h/L t1/2 2.8 ± 0.8 Vd (L) 13.1 ± 11.1 CLtot 61.1 ± 55.2 mL/min CLdial 80.1 ± 7.7 mL/min	Ampicillin/sulbactam is eliminated by ED Current dosing recommendations from patients undergoing IHD (3 g every 24 h) would cause a significant underdosing of the drug in patients treated with ED Ampicillin/sulbactam concentrations exceeded MIC90 values of *Enterobacteriaceae*, such as *Escherichia coli* or *Klebsiella pneumoniae* (MIC90 < 2.0 mg/L) or *Enterococcus faecalis* (MIC90 = 2.0 mg/L), only for 8 h (approximately 30% of the dosing interval for patients on intermittent hemodialysis) after start of infusion. A dosage of 3 g every 12 h in patients undergoing ED does not lead to a significant accumulation of the drug
Burkhardt et al. [84]	To evaluate PK of ertapenem, with once-daily dosing, in critically ill patients with anuric acute renal failure undergoing EDD	Ertapenem	Prospective, open-label study 6 adult ICU patients undergoing EDD treated with 1 g ertapenem as a single intravenous dose Blood samples were collected before ertapenem infusion and 0.5, 1, 2, 4, 6, 8 h after the end of the infusion and also 2, 4, 6, and 8 h after the start of EDD. Additional blood samples were drawn pre- and post-dialysis in order to calculate the dialyzer clearance. To study post-EDD PK, samples were drawn 0.5, 1, 3, and 8 h after the end of EDD	Cmax 81.3 ± 12.1 mg/L AUC0-inf 687.4 ± 212.0 mg.h/L T1/2off 18.9 ± 5.4 h T1/2on 6.7 ± 0.4 h Vd (L) 15.9 ± 3.2 CLoff 19.3 ± 11.4 mL/min CLdial 49.5 ± 10.9 mL/min T > MIC was 100% (MIC90 ≤ 1 mg/L) and 85% (MIC90 ≤ 2 mg/L)	1 g ertapenem per day to critically ill patients with ARF in the ICU that undergo EDD is necessary to ensure optimal free concentrations of ertapenem. A reduction of the dose is not supported by our data. Further dosing recommendations for patients with renal failure in the ICU treated with such effective modes of renal replacement therapy should be developed to avoid excess mortality due to under-dosing of life-saving medication
Tamme et al. [85]	To describe the PK of piperacillin and tazobactam during extended high volume hemodiafiltration to define optimal dosing	Piperacilin-tazobactam	Prospective, observational study 10 adult ICU patients with sepsis and AKI requiring CRRT A single dose of 4000 mg of piperacillin and 500 mg tazobactam was administered as a 30-min intravenous infusion 1 h after the start of HVHDF Blood samples of 4 mL were collected before and immediately after the end of piperacillin/tazobactam infusion and 60, 90, 120, 150, 180, 240, 300, 360, 420, and 480 min after the start of drug administration	The plasma concentration–time profiles of piperacillin and especially tazobactam demonstrated high interindividual variability For piperacilin CL (range) 6.9 L/h (6.1–7.9) Vd central compartment (range) 9.0 L (7.4–11.0) Vd peripheral compartment (range) 11.2 L (8.9–14.2) For tazobactam CL (range) 5.1 L/h (4.1–6.3) Vd central compartment (range) 8.6 L (6.9–10.7) Vd peripheral compartment (range) 8.9 L (6.6–12.0) Using Monte Carlo simulation, the probability of 100% fT > MIC target attainment for piperacillin/tazobactam 4.5 g dosed every 6 and 8 h as 4-h infusion were 88.6% and 61.0%, respectively, for MIC 16 mg/l	Application of extended HVHDF for the treatment of AKI in septic shock patients results in considerable clearance of piperacillin and tazobactam Piperacillin/tazobactam doses of 4.5 g, administered every 8 h as 0.5-h infusion during HVHDF, ensured more than 80% probability of attaining the 50% fT > MIC target for intermediately susceptible bacteria (MIC 16 mg/l) While aiming for 100% fT > MIC of 16 mg/l, increasing doses to 4.5 g every 6 h and prolonging the infusion time to 4 h would be necessary

*AKI* acute kidney injury, *ARF* acute renal failure, *CLdial* dialysis clearance, *CLoff* drug clearance without dialysis, *EDD* extended daily dialysis, *ICU* intensive care unit, *SD* standard deviation, *SLED* sustained low-efficiency dialysis, *T1/2off* half-life before/after EDD, *T1/2on* half-life during EDD, *Vd* volume of distribution

Globally, we recommend not reducing standard antibiotic dosage since no drug accumulation was found in the available literature and to maintain continuous or prolonged infusion in critically ill patients on CRRT, SLED, or EDD, especially for the treatment of multidrug-resistant bacteria. Although usually not available in clinical routines, a therapeutic drug monitoring (TDM)-guided strategy has potential benefit to ensure appropriate antibiotic therapeutic targets.

Extracorporeal membrane oxygenation (ECMO) has become an essential tool for severe cardiorespiratory failure in critically ill patients. It is thought to introduce additional confounding factors to the already altered PK properties of beta-lactams in this subset of patients. Sequestration of antibiotics in the ECMO circuit and the associated systemic inflammation can further increase the antibiotic Vd and reduce clearance [74, 97–99]. However, very few in vivo studies have been performed in this subset of patients (Table 3). Globally, they show no significant statistical variation in Vd and clearance, but while probability of target attainment (PTA) with standard ICU dosage regimens was achieved when treating for highly susceptible Gram-negative bacteria, antibiotic concentrations were below those desired to treat more resistant strains.

**Table 3:** PK/PD studies of beta-lactams in patients with extracorporeal membrane oxygenation

Study	Endpoints	Antibiotic	Design	Results	Conclusions
Donadello et al. [100]	To investigate whether ECMO could alter the pharmacokinetics of meropenem and piperacillin/tazobactam in ICU patients	Meropenem Piperacilin/tazobactam	Retrospective, case-control study in 67 ICU patients Antibiotics daily dosing was done according to renal dosing Beta-lactam plasma concentrations were measured T2 and just before administration of the subsequent dose T0 TDM results in ECMO patients (VV or VA) were matched (1:1) to TDM results of non-ECMO patients (total 41 TDM matches) according to the following criteria: drug regimen; renal function (same ClCr or, if on CRRT, same CRRT intensity with an acceptable difference of 10%); total body weight; SOFA score at the time of treatment initiation; age	For both antibiotics, there were numerical differences but with no statistical significance in V_d_, t1/2 and CL between ECMO patients and controls The proportions of insufficient (13/41 vs12/41), adequate (15/41 vs 19/41), and excessive (13/41 vs 10/41) drug concentrations were similar in ECMO and control patients	PK parameters and TDM results were not significantly altered in ECMO patients compared with control ICU patients Almost 30% of the overall TDM results were associated with insufficient antibiotic concentrations to optimally treat *P. aeruginosa*
Shekar et al. [101]	To describe single-dose meropenem PK during ECMO using critically ill patients with sepsis and not receiving ECMO as controls	Meropenem	Open-label, descriptive, matched-cohort PK study Adult ICU patients on ECMO (no RRT *n* = 6; on RRT *n* = 5) and controls (no renal dysfunction *n* = 5; on RRT *n* = 5) Meropenem doses in ECMO: 1 g 8/8 h (*n* = 8); 1.5 g 8/8 h (*n* = 2) and 2 g bolus; and 1 g 8/8 h (*n* = 1) Controls: 1.5 g bolus and 1 g 8/8 h (no renal dysfunction); 1 g 8/8 h	Controls vs ECMO Cmax: 65.4 (58.7–74.4) vs 55.3 (37.8–60.4) mg/L Cmin: 4.2 (0.0–5.7) vs 7.2 (4.0–17.2) mg/L Vd: 0.45 ± 0.17 vs 0.41 ± 0.13 L/kg, *P* = 0.21 Clearance: 7.9 ± 5.9 vs 11.7 ± 6.5 L/h, *P* = 0.18 ECMO patients, trough concentrations > 2 mg/L were achieved in all patients. Through concentrations > 8 mg/L (targeting less susceptible microorganisms) were achieved in only 8 out of 11 patients, 5 of them being on RRT	Standard meropenem dosing (1 g IV 8-hourly) as an intermittent bolus infusion in ECMO patients is likely to result in drug concentrations sufficient to treat highly susceptible Gram-negative pathogens However, when treating less susceptible *P. aeruginosa* (MIC_90_ 8 mg/L) and *Acinetobacter* species (MIC_90_ 16 mg/L) higher meropenem doses may have to be considered
Welsch et al. [102]	To report the cases of two patients on VV ECMO for refractory ARDS following lung transplantation and treated empirically with imipenem	Imipenem	Case report Imipenem 1 g every 6 h Serum and mini-BAL samples (native and transplant lung) collected at steady state after 2 days of therapy immediately before the fifth drug dose *Enterobacter cloacae* was isolated from the respiratory sample of patient 1 and *Klebsiella pneumoniae* was isolated from the respiratory sample of patient 2 MIC of the two isolated strains were 0.125 and 0.25 mg/L, respectively	BAL concentrations were undetectable (< 0.5 mg/L) Serum T > MIC of both microorganisms was 100% Considering more resistant microorganisms, such as *P. aeruginosa* with MIC > 2 mg/L, the probability of achieving a fractional time above MIC > 50% or 100% was also high There was great variability in the residual serum concentration of imipenem between the two patients	An elevated dosing regimen (4 g/24 h) is more likely to optimize drug exposure, and therapeutic drug monitoring is recommended

*ARDS* acute respiratory distress syndrome, *BAL* bronchial-alveolar lavage, *CL* clearance, *ClCr* creatinine clearance, *CRRT* continuous renal replacement therapy, *ECMO* extracorporeal membrane oxygenation, *ICU* intensive care unit, *MIC* minimal inhibitory concentration, *RRT* renal replacement therapy, *SOFA* sequential organ failure assessment, *T > MIC* percentage of time above minimal inhibitory concentration, *T0* 0 h after the start of infusion, *T2* 2 h after the start of infusion, *TDM* therapeutic drug monitoring, *V*_*d*_ volume of distribution, venous-arterial, *VV* venous-venous

## Longer exposure regimens: continuous infusion, extended infusion, or reduced-interval dosing

The duration of infusion of beta-lactams has been shown to influence their fT > MIC. Improved PD profiles of beta-lactams may be obtained by longer exposure with more frequent dosing, extended infusions, or continuous infusions. Several studies reported PD benefits for target attainment of extended and continuous infusions, especially considering highly resistant bacterial strains, even using smaller daily doses [1, 2, 36, 41, 103–143]. However results are conflicting concerning decreased mortality and bacteriological and clinical cure rates [144]. A sub-analysis from the DALI study compared intermittent bolus vs prolonged infusions of beta-lactams in patients with respiratory infection and concluded that patients receiving beta-lactams via prolonged infusion demonstrated significantly better 30-day survival [145].

Falagas et al. [114] conducted a meta-analysis of 14 studies comparing continuous and short-term infusion of carbapenems and piperacilin-tazobactam, involving 1229 patients. Mortality was lower among patients receiving extended or continuous infusion of carbapenems or piperacillin/tazobactam compared to those receiving short-term infusion (risk ratio (RR) 0.59, 95% confidence interval (CI) 0.41–0.83). Patients with pneumonia who received extended or continuous infusion had lower mortality than those receiving short-term infusion (RR 0.50, 95% CI 0.26–0.96) [114].

An interesting retrospective study by Huang et al. [120] reviewed 68 neurosurgical patients with post-operative intracranial infections treated with 4 g/day cefepime over 24 h as a continuous infusion (CI; *n* = 34) or 2 g every 12 h as intermittent infusion (II; n = 34). CI controlled the intracranial infection more rapidly and effectively than II (6.6 ± 1.9 days vs 7.8 ± 2.6 days; *P* = 0.036). PD targets were more achievable with CI: for plasma cefepime concentrations, the percentage fT > MIC in the CI group was higher than in the II group (for MICs of 8 μg/mL, 100% vs 75%, respectively). For cerebral spinal fluid (CSF) cefepime concentrations, the percentage fT > MIC in the CI group was higher than in the II group (for MICs of 4 μg/mL and 8 μg/mL, 83.3% and 75% vs 25% and 0%, respectively) [120].

De Waele et al. [27] reviewed 343 patients from 68 ICUs across ten countries and concluded that use of intermittent infusion was the most significant factor associated with target non-attainment, for both 50% and 100% fT > MIC. Other risk factors for target non-attainment were ClCr, recent surgery, and timing from initial antibiotic therapy and sampling. However, the type of infusion was such a significant covariate in the model that it eliminated the effects of other variables [27].

## Site of infection

Usually drug concentrations in blood are used to determine PD parameters, such as percentage of time drug levels exceed the MIC and peak drug AUC/MIC level, due to the relative accessibility of this body fluid. Because infection usually occurs at extravascular sites, the use of drug concentrations in blood is only satisfactory if blood levels are an adequate surrogate for levels at the site of infection [13]. In septic shock, blood misdistribution in the microcirculation might decrease antibiotic concentration at the infection site [1].

Boyadjiev et al. [146] studied ertapenem penetration into muscle in mechanically ventilated patients and concluded that average muscle free-ertapenem concentrations were above the MIC values of targeted pathogens except in a few patients. Karjagin et al. [147] evaluated the PK/PD relations of meropenem in plasma and peritoneal fluid by microdialysis and showed that area under the concentration–time curve was lower in peritoneal fluid than in plasma, concluding that in patients with severe peritonitis associated with septic shock, a dosing regimen of 1 g infused over 20 min every 8 h is sufficient against susceptible bacteria, but not always against intermediately susceptible bacteria. Also, beta-lactam PK is variable between plasma and subcutaneous interstitial fluid in septic patients [148]. Thus, prediction of microbiological outcome based on concentrations in plasma results in overestimation of antimicrobial activity at the site of infection.

Special anatomic barriers (e.g., brain, eye, and prostate) can result in drug levels being much lower than free drug levels in plasma [13]. The combination of tight junctions and active transport systems that form the blood–brain barrier creates a substantial impediment to the penetration of most antibiotics into the CSF. However, the presence of inflammation within the meninges significantly alters the permeability of the blood–brain barrier, increasing CSF exposure for the majority of antibiotics [20]. For meningitis, CSF levels are appropriate for determination of PD parameters.

Very few studies have investigated PK/PD issues in the CSF (Table 4). Five case reports, one randomized clinical trial in a paediatric population, and three prospective observational studies found good probability of target attainment for susceptible strains but standard dosing may not be optimal for less susceptible strains. Prolonged and/or continuous infusion is of benefit in the attempt to achieve PD targets. No data regarding intermittent versus continuous CSF ventricular drainage were found and conceptually these two types of drainage may alter the beta-lactam PK profile.

**Table 4:** PK/PD studies of beta-lactams in cerebral spinal fluid

Study	Endpoints	Antibiotic	Design	Results	Conclusions
Goldwater et al. [149]	To evaluate antibiotic CSF penetration and antimicrobial efficacy	Ceftriaxone Cefotaxime	Randomised, open, comparative trial 120 paediatric patients with meningitis, 33 with repeated lumbar puncture at different times CRO 100 mg/kg once daily; CTX 50 mg/kg every 6 h	All 33 repeated lumbar punctures were sterile The lowest CSF level recorded (0.45 μg/mL for CTX) was 45 times the MIC (0.01 μg/mL). The highest levels (24–35 μg/mL for CRO) were up to 8750 times the MIC of the patient’s causative organism	Antibiotic levels achieved in CSF were therapeutic, being well above the MIC for all organisms encountered CSF cell count had no apparent influence on antibiotic levels
Lonsdale et al. [150]	To illustrate issues in the management of CSF antibiotic concentrations	Meropenem	Case report Neurosurgical patient with external ventricular drain-related ventriculitis	Adequate plasma through concentrations achievable after increasing dosing to 2 g, four times daily CSF concentrations of meropenem were similar to those seen in plasma There were variations in CSF drug penetration	Achieving CSF therapeutic antibacterial concentrations in neurosurgical critically ill patients is difficult Standard antibacterial prescription is potentially flawed in this setting, suggesting the need for therapeutic drug monitoring
Abdul-Aziz et al. [151]	To report the difficulty in achieving and maintaining target antibiotic exposure in critically ill patients with deep-seeded infections	Flucloxacillin	Case report Critical care patient with CNS infection	Trough plasma concentrations were below the MIC; CSF concentrations were undetectable (intermittent doses 2 g 6/6 h) With continuous infusion and increasing the dose to 20 g daily, the plasma and CSF levels became detectable, albeit lower than the predefined targets	Antibiotic pharmacokinetics may be significantly altered in critically ill patients Applying continuous infusion and monitoring plasma and CSF levels is of significance to optimize antibiotic delivery
Cies et al. [152]	To describe the pharmacokinetics of continuous-infusion of meropenem	Meropenem	Case report. Paediatric patient with ventriculitis	Serum levels were 12 μg/mL at 2 h and “undetectable” at 4 h, with CSF levels of 1 and 0.5 μg/mL at 2 and 4 h, respectively (MIC < 0.25) On continuous infusion, serum, and CSF levels were noted to be 13 and 0.5 μg/mL, respectively	The continuous-infusion dosing regimen allowed for 100% probability of target attainment in the serum and CSF and a successful clinical outcome
Dahyot-Fizelier et al. [153]	To describe brain distribution of cefotaxime by microdialysis in patients with acute brain injury	Cefotaxime	Observational, prospective study 5 ICU patients with acute brain injury, treated for lung infection Cefotaxime 2 g 8/8 h	Mean AUC_brain_/AUC_plasma_ ratio was 26.1 ± 12.1% Unbound cefotaxime brain concentrations were much lower than corresponding plasma concentrations Simulated brain concentration at two dosage regimens (used for treatment of meningitis) showed T > MIC higher than 90% of the dosing interval for both dosing regimens (4 g every 6 h or 8 h) for susceptible strains and only for 4 g every 6 h for resistant ones	There is limited brain distribution of cefotaxime Higher cefotaxime dosage (4 g 6/6 h) is required to treat meningitis with resistant bacterial strains
Morita et al. [154]	To assess the efficacy, safety, and concentration of meropenem in cerebrospinal fluid	Meropenem	Observational, prospective study 5 adult ICU patients with meningitis Meropenem 2 g 8/h (duration of infusion was variable from 0.5 to 2 h) CSF and blood were obtained pre-treatment and on days 3, 7, 14, and 21	Concentrations in cerebrospinal fluid ranged from 0.27 to 6.40 μg/mL up to 8.47 h and were over 1 μg/mL 3 h after starting meropenem infusion The CSF/plasma concentration ratio ranged from 0.008 to 0.013, 0.011 to 0.953, and 0.633 to 1.821, respectively, within 2 h, 2–6 h, and after 6 h from the start of drug infusion The relationship of CSF concentration to CSF cell counts, CSF/plasma glucose ratio, and CSF protein concentration, respectively, was statistically correlated with CSF cell counts and CSF protein concentration, and inversely correlated with CSF/plasma glucose ratio	Concentration of meropenem in CSF exceeded the minimal inhibitory concentration for the pathogens involved (penicillin sensitive *S. pneumoniae* and *S. salivarius*; methicillin-sensitive *Staph aureus*) No serious adverse event and no discontinuation of treatment occurred The meropenem concentration in the CSF can be expected to be high because of the presence of inflammation
Tsumura et al. [155]	To examine PK and PD of meropenem in cerebrospinal fluid	Meropenem	Observational, prospective study 6 neurosurgical patients Meropenem (0.5 g every 8 h) was administered during 0.5 h. Lumbar CSF and venous blood samples were obtained at 0.5–16 h after the start of the first infusion	Penetration into the CSF with the AUC ratio was 0.10 ± 0.03 (mean ± SD) A dosage of 0.5 g q8h achieved a > 90% PTA (50% of the T > MIC), and 1 g q8h was needed for a > 90% PTA (100% of the T > MIC) for susceptible isolates For *P. aeruginosa*, 2 g q8h achieved a lower PTA	Less susceptible bacterial CNS infections may not be optimized with standard meropenem dosage
Nicasio et al. [156]	To describe the use and cerebral spinal fluid penetration of a prolonged infusion meropenem regimen in a patient with *Serratia marcescens* meningitis	Meropenem	Case report Adult patient with meningitis and epidural abscess Meropenem 2 g q8h, 3-h infusion	The prolonged (3 h) infusion regimen of 2 g 8 h resulted in concentrations in both serum and CSF above the MIC of 0.047 μg/mL, for 100% of the dosing interval CSF penetration was 6.4%	The use of a high-dose prolonged infusion of meropenem resulted in adequate exposure at the site of infection and a successful clinical response At follow-up, the patient had completed a 4-week course without relapse or adverse events
Frasca et al. [157]	To describe PK–PD profile of cefotaxime in the CSF	Cefotaxime	Case report Adult ICU patient with TBI Cefotaxime 4 g q8h, 30-min infusion Microdialysis was performed on day 4, after the 12th dose	Unbound plasma Cmax was 118.8 μg/mL CSF Cmax was 11.4 μg/mL T > MIC in the brain were, respectively, 78% (6.2 h) and 46% (3.7 h) for MIC values of 2 and 4 μg/mL	ECF brain concentrations indicate that an adequate exposure to cefotaxime is achieved in prevention and treatment of most CNS infections with the standard dosage regimen
Wang et al. [158]	To explore whether there is increased CSF penetration of cefoperazone/sulbactam when thee blood–brain barrier is impaired following craniotomy; and whether extended infusion time affects drug concentrations	Cefoperazone/sulbactam	Observational, prospective study Dosing was 3.0 g in a 3-h infusion every 6 h, after craniotomy Venous blood and CSF were collected before the start of drug administration and at hour 1, 2, 3, 4, 6, 12, 15, 16, and 18 after administration 8 neurosurgical adult patients enrolled	CSF penetration: Peak concentrations (CSF/serum): 8.6% ± 7.2% for cefoperazone and 13.5% ± 11.9% for sulbactam Trough concentrations (CSF/serum): 13.4% ± 5.3% for cefoperazone and 106.5% ± 87.5% for sulbactam Ratio of the AUC of CSF and serum: 14.5% for cefoperazone and 22.6% for sulbactam Cefoperazone serum concentrations achieved > 50%T > MIC for *Pseudomonas* and *Acinetobacter* (MIC_90_ 64 mg/L) and 100%T > MIC for more susceptible bacteria CSF cefoperazone T > MIC% was almost 100% (*Escherichia coli* MIC50) and T > MIC% was more than 50% (*Acinetobacter baumannii* MIC50) Sulbactam serum concentrations achieved > 50%T > MIC for *Acinetobacter* (MIC_90_ 16 mg/L) CSF sulbactam concentrations did not reach the level of MIC_50_ of 8 mg/L for *Acinetobacter*	If cefoperazone/sulbactam single infusion time is extended to 3 h, the serum drug concentration achieved the PK/PD standard of > 50%T > MIC (MIC_90_ 64 mg/L) It is very difficult to achieve this PK/PD standard in the CSF, and a higher dose might be needed to treat intracranial infections Destruction of the blood–brain barrier after craniotomy can increase the CSF concentration to a certain extent

*AUC* area under the curve, *CNS* central nervous system, *CRO* ceftriaxone, *CSF* cerebral spinal fluid, *CTX* cefotaxime, *ICU* intensive care unit, *MIC* minimal inhibitory concentration, *MIC50* minimal inhibitory concentration for 50% of isolates, *PTA* probability of target attainment, *SD* standard deviation, *T > MIC* percentage of time above minimal inhibitory concentration, *TBI* trauma brain injury

There is very sparse data on possible surrogate central nervous system penetration factors for beta-lactams, so no conclusions can be made. We recommend to use higher than standard dosing, preferably with continuous or prolonged infusions, especially when treating less susceptible bacterial strains. Toxicity did not increase at increased doses. Finally, none of these studies addressed clinical outcome.

Though there are PK models of plasma concentrations of beta-lactams specifically for the critically ill population with pneumonia, it is suggested that epithelial lining fluid (ELF) concentrations are important determinants of efficacy of treatment of bacterial pneumonia. ELF-to-serum penetration ratios may vary widely among beta-lactams [13, 20, 159]. The impact of infection on their penetration into ELF in humans is unknown [159], though some reports state that ELF penetration increases in acute lung injury [160].

Only a few studies have investigated beta-lactam PK/PD issues in critically ill patients with pneumonia (Table 5) and in only seven of them were ELF drug concentrations measured. A standard dosage of beta-lactams derived from healthy patients’ PK profiles may be insufficient for treatment of critically ill patients with pneumonia, especially when caused by multidrug-resistant pathogens. Continuous or prolonged infusions and higher than standard doses improve the PD profiles of these antibiotics. This is very important to achieve an adequate PD profile when treating less susceptible bacterial strains. Therapy drug monitoring would be extremely helpful in this setting.

**Table 5:** PK/PD studies of beta-lactams in bronchial-alveolar lavage

Study	Endpoints	Antibiotic	Design	Results	Conclusions
Kikuchi et al. [52]	Compare the PK/PD parameters of biapenem in bronchial ELF given as 0.5-h and 3-h infusions	Biapenem	Prospective, non-blinded, crossover study 6 healthy adult volunteers	The percentage (mean ± SD) of T > MIC in bronchial ELF ranged from zero (MIC 4 μg/mL) to 34.6% ± 5.2% (MIC 0.25 μg/mL) after the 0.5-h infusion and from 5.1% ± 5.6% (MIC 4 μg/mL) to 52.2% ± 17.0% (MIC 0.25 μg/mL) after the 3-h infusion	A 3-h infusion of biapenem tended to produce a higher T > MIC in bronchial ELF, as well as in plasma, than a 0.5-h infusion These results support the use of prolonged infusions for successful treatment of lower respiratory tract infections based on PK/PD parameters in bronchial ELF
Rodvold et al. [159]	To define the exposure targets in lung associated with good microbiological activity in a murine model To determine drug penetration into ELF in humans	Ceftobiprol	Prospective, observational study Pre-clinical, murine model Clinical study with 24 healthy volunteers 500 mg every 8 h, 2-h infusion regimen	Murine model: for cell kills of 1 and 2 log_10_ CFU/g, total drug must be present in ELF at a concentration in excess of the MIC of 12.9% and 24% of a 24-h interval, respectively. ELF penetration was 69% (median) Mean ELF penetration in human volunteers was 25.5% (median, 15.3%; interquartile range, 7.9% to 30.4%) Target attainment falls below 90% for a cell kill of 2 log_10_ CFU/g at a MIC of 1.0 mg/L and for a 1 log_10_ CFU/g cell kill at a MIC of 2.0 mg/L	Ceftobiprole penetrated into ELF very differently in humans compared to mice For seriously ill patients, particularly in the ICU, higher doses or longer infusion times (to prolong the time > MIC), or both, will be required to comfortably ensure a 90% target attainment for seriously ill patients with MRSA pneumonia
Conte et al.	Determine the plasma and intrapulmonary ELF and AC pharmacokinetic parameters of intravenously administered meropenem	Meropenem	Prospective, observational Four doses (8/8 h) of 0.5 g, 1.0 g or 2.0 g were administered intravenously to 20, 20, and 8 healthy adult subjects, respectively	C_max_, AUC, T_1/2_: 0.5 g group: serum 25.8 ± 5.8 μg /mL, 28.57μg h/mL, 0.77 h; ELF 5.3 ± 2.5 μg/mL, 12.27 μg h/mL, 1.51 h; AC 1.0 ± 0.5μg/mL, 4.30 μg h/mL, 2.61 h 1 g group: serum 53.5 ± 19.7 μg /mL, 55.49μg h/mL, 1.31 h; ELF 7.7 ± 3.1μg /mL, 15.34 μg h/mL, 0.95 h; AC 5.0 ± 3.4 μg/mL, 14.07 μg h/mL, 2.17 h 2 g group: serum 131.7 ± 18.2μg/mL, 156.7 μg h/mL, 0.89 h T ≥ MIC (MIC90 values of 0.12–4μg/mL): 0.5 g group: serum 28–78%, ELF 18–100%, AC 0–100% 1 g Group: serum 45–100%, ELF 25–88%, AC 24–100%	The prolonged T > MIC90 and high intrapulmonary drug concentrations following every 8 h administration of 0.5–2.0 g doses of meropenem are favorable for the treatment of common respiratory pathogens.
Boselli et al. [161]	Determine the steady-state serum and alveolar concentrations of piperacillin/tazobactam administered in continuous infusion (12/1.5 g/day or 16/2 g/day) at various degrees of renal failure (terminal renal failure excluded)	Piperacilin/tazobactam	Prospective, open-label, comparative, single center 40 ICU patients with ventilator-associated pneumonia Samples collected after 2 days of treatment	Median (interquartile) serum and alveolar piperacilin concentrations No/mild renal failure (creatinine clearance ≥ 50 mL/min) Serum 25.3 mg/L (23.1–32.6) and alveolar 12.7 mg/L (6.7–18.0) for 12/1.5 g/day Serum 38.9 mg/L (32.9–59.6) and alveolar 19.1 mg/L (14.0–21.5) for 16/2 g/day Moderate/advanced renal failure (creatinine clearance < 50 mL/min) Serum 102.4 mg/L (97.4–112.6) and alveolar 44.1 mg/L (33.4–48.3) for 12/2 g/day Serum 135.3 mg/L (119.5–146.2) and alveolar 54.9 mg/L (45.2–110.3) for 16/2 g/day Alveolar penetration was 40–50% for piperacillin and 65–85% for tazobactam There was a positive linear relationship between ELF and serum concentration in both patients receiving 12/1.5 g/day (*r* = 0.8437, *P* < 0.0001) and 16/2 g/day (*r* = 0.7935, *P* < 0.0001) T > MIC was 100% (assuming MIC > 16 mg/L) in both serum and ELF in all patients with moderate/advanced renal failure; in patients with no/mild renal failure this threshold was not reached in 6 patients with 12/0.5 g dosing and 4 patients with 16/2 g dosing	The administration of daily continuous infusions of P/T 12/1.5 g or even 16/2 g might provide insufficient alveolar concentration to eradicate high-risk pathogens with high MICs such as multi-drug resistant *P. aeruginosa* in patients with no or mild renal insufficiency A linear relationship between alveolar and serum piperacillin concentration was observed in this study, with ELF piperacillin concentration being 40–50% of corresponding serum values
Cousson et al. [162]	Compare continuous vs intermittent administration of drug Determine average concentration in ELF Determine the fraction of time, during the first 48 h of treatment, where serum concentrations remained above the 20 mg/L plasma threshold	Ceftazidime	Single-center, controlled, randomized trial in two parallel groups comparing two modes of administration: group A, loading dose 20 mg/kg + 60 mg/kg/day; group B, 20 mg/kg over 30 min every 8 h 34 adult ICU patients with ventilator-associated pneumonia due to Gram-negative bacilli. The mean MIC of ceftazidime for *P. aeruginosa* was estimated at 2 mg/L. The target threshold value for ceftazidime in the ELF was thus fixed at 8 mg/L, with a corresponding serum threshold of 20 mg/L	Plasma *T* > 20 mg was 100% in group A versus 46% in group B (*P* < 0.003) In the ELF, the median concentration was 12 mg/L in group A versus 6 mg/L in group B (*P* < 0.08) A threshold of 8 mg/L in the epithelial lining fluid was achieved twice as often in group A as in group B	Continuous infusion presents advantages in terms of PD and predictable efficacy in patients presenting ventilator-associated pneumonia
Burkhardt et al. [163]	Determine in vivo penetration into LT, ELF, and AC after 1 g of ertapenem (infusion period 30 min) for perioperative prophylaxis	Ertapenem	Single-center, prospective, observational of 15 patients undergoing thoracotomy BAL performed at 1, 3, and 5 h after ertapenem infusion Plasma concentration collected up to 24 h after infusion LT collected at time of lung extraction	Mean concentrations in plasma, ELF, and AC were: at 1.0 h, 63.1, 4.06, 0.004 mg/L; at 3.0 h, 39.7, 2.59, 0.003 mg/L; at 5.0 h, 27.2, 2.83, 0.007 mg/L Mean (range) concentration in LT was 7.60 (2.5–19.4) mg/kg tissue, 1.5 to 4.5 h after infusion and LT plasma concentration ratio was 23.6 ± 12.3% Mean level of ELF penetration was 7.48% ± 8.17%, and the highest degree of penetration was recorded at 5 h after infusion (mean ± SD, 9.40% ± 10.7%) A 1-g dose, once daily, results in drug plasma concentrations higher than MICs of most community-acquired respiratory pathogens during the entire dosing interval Mean concentrations in ELF exceeded MIC90 values of highly or moderately penicillin-sensitive *Pneumococci* and of *H.* *influenzae* within 1 to 5 h after start of infusion and were partly below the MIC90 of penicillin-resistant *Pneumococci*	These results, combined with the reported MIC_90_ of most CAP bacteria, support the previously observed clinical efficacy of ertapenem in the treatment of community-acquired pneumonia
Boselli et al. [164]	Determine the steady-state serum and ELF concentrations of unbound ertapenem administered once daily to critically ill patients with early-onset ventilator-associated pneumonia	Ertapenem	Prospective, open-label study in an intensive care unit 15 patients with VAP received 1-h intravenous infusions of 1 g ertapenem once daily Samples obtained at steady-state, after 2 days of therapy ELF obtained by mini-BAL	Median (interquartile range) C_max_, C_12_, and C_min_ concentrations (mg/l) 1, 12, and 24 h after the end of infusion were: 30.3 (27.1–37.8), 4.8 (3.9–6.4), and 0.8(0.5–1.2) in serum and 9.4 (8.0–10.7), 2.0 (1.1–2.5), and 0.3 (0.2–0.4) in ELF, respectively Median free percentage penetration in ELF approximately 30–40%	Concentrations exceeding the MIC90 values of most of the causative pathogens (0.25–2 mg/l for *S. pneumoniae*, 0.06–0.125 mg/l for *Haemophilus influenzae*, 0.25–0.5 mg/l for oxacillin-susceptible *S. aureus*, and 0.03–0.125 mg/l for *Enterobacteriaceae* and anaerobes 0.5–1 mg/l was encountered in early-onset VAP during 50–100% time 1 g intravenous ertapenem once daily should be effective for early-onset VAP ICU patients with no known risk factors for multidrug-resistant pathogens
Boselli et al. [165]	To determine the steady-state plasma and ELF concentrations of ceftazidime administered in continuous infusion to critically ill patients with severe nosocomial pneumonia	Ceftazidime	Prospective, open-label study. 15 adult patients with severe nosocomial bacterial pneumonia on mechanical ventilation Administration of 30 min infusion of 2 g ceftazidime followed by continuous infusion 4 g over 24 h Blood and mini-BAL samples collected by the third day of antibiotic therapy (8:00 am, 12:00 pm, and 6:00 pm)	The mean ± SD steady-state plasma and ELF concentrations in continuous infusion were 39.6 ± 15.2 g/mL and 8.2 ± 4.8 g/mL, respectively, showing a mean ± SD percentage penetration of ceftazidime into ELF of 20.6 ± 8.9%	The administration of the applied dose in critically ill patients with severe nosocomial pneumonia provides concentrations in excess of the MIC of many susceptible organisms over the course of therapy in both serum and ELF. However, for some pathogens such as *P. aeruginosa*, higher doses of ceftazidime should be administered, or another agent should be used in combination
Felton et al. [166]	To assess plasma and intra-pulmonary PK of piperacillin/tazobactam in critically ill patients Quantify pulmonary penetration Identify factors that may influence pulmonary penetration	Piperacilin/tazobactam	Prospective, open label, single arm study 18 ICU adult patients with pneumonia Administration of 4/0.5 g infusion every 8 h daily Samples collected at steady-state (mean of 8.8 doses (range 2–16). Non-directed bronchial lavage was performed for ELF sampling	Median piperacilin and tazobactam pulmonary penetration ratio was 49.3% and 121.2% respectively. ELF protein/plasma protein ratio, as a surrogate measure of lung permeability, was found to have a statistically negative correlation between piperacilin pulmonary penetration ratio and pulmonary permeability. There was no statistically significant correlation for tazobactam 14–18% of patients will have suboptimal drug exposure (ELF 50%T > MIC) when infected with a “susceptible” (MIC < 16 mg/L) organism Piperacilin and tazobactam plasma concentrations do not precisely predict ELF concentrations of both drugs	Current piperacilin-tazobactam regimens is inadequate for effective treatment and suppression of emergence of antimicrobial resistance in an unacceptably high proportion of critically ill patients, especially those with pneumonia resulting from infection with a less susceptible organism
Boselli et al. [167]	To determine the steady-state plasma and ELF concentrations of cefepime administered in continuous infusion in critically ill patients with severe bacterial pneumonia	Cefepime	Prospective, open-label study 20 ICU adult patients with severe nosocomial bacterial pneumonia All subjects received a 30-min intravenous infusion of cefepime 2 g followed by a continuous infusion of 4 g over 24 h Samples were collected after 48 h of therapy. Blood and mini BAL samples were collected at 8:00 am, 12:00 pm, and 6:00 pm	Mean ± SD steady-state plasma and ELF concentrations were 13.5 ± 3.3 g/mL and 14.1 ± 2.8 g/mL, respectively, with a mean percentage penetration into epithelial lining fluid of about 100%	Administration of cefepime in continuous infusion in critically ill patients with severe nosocomial pneumonia appears to optimize the pharmacodynamic profile of this beta-lactam by constantly providing concentrations in excess of MIC of most of the susceptible organisms over the course of therapy in both serum and ELF For some pathogens such as *Acinetobacter* spp. and *P. aeruginosa* higher doses should be administered
Boselli et al. [168]	To determine the steady-state plasma and epithelial lining fluid concentrations of piperacillin/tazobactam administered to critically ill patients with severe bacterial pneumonia	Piperacilin/tazobactam	Prospective, open label study 10 ICU adult patients with severe nosocomial pneumonia 30-min intravenous infusion 4/0.5 g every 8 h Samples were obtained at steady-state, after 2 days of treatment. Blood samples were collected at three predetermined time points at 7:00 am (trough), 8:00 am (peak), and 12:00 pm (intermediate) Mini BAL was performed simultaneously to blood sampling at 12:00 pm	Mean ± SD steady-state plasma trough, peak, and intermediate concentrations were 8.5 ± 4.6 μg/mL, 55.9 ± 21.6 μg/mL, and 24.0 ± 13.8 μg/mL for piperacillin, and 2.1 ± 1.0 μg/mL, 4.8 ± 2.1 μg/mL, and 2.4 ± 1.2 μg/mL for tazobactam Mean ± SD steady-state intermediate ELF concentrations were 13.6 ± 9.4 μg/mL for piperacillin and 2.1 ± 1.1 μg/mL for tazobactam, respectively Mean percentage penetration of piperacillin and tazobactam into ELF was 56.8% and 91.3%, respectively	Treatment of severe nosocomial pneumonia with a regimen of P/T 4/0.5 g every 8 h might provide insufficient concentrations into lung tissue to exceed the MIC of many causative pathogens
Lodise et al. [169]	To describe the PD profile of cefditoren in plasma and ELF	Cefditoren	Open, noncontrolled, dual-center, phase I study 24 adult patients with scheduled bronchoscopy Single oral dose 400 mg cefditoren Three sampling time windows (1–2 h, 2–3 h, or 3–4 h post-administration)	Plasma/ELF concentrations (mg/L)/penetration ratios 1–2 h: 1.78 ± 1.27/0.39 ± 0.21/38.1 ± 50.1% 2–3 h: 1.33 ± 0.95/0.34 ± 0.25/23.2 ± 18.1% 3–4 h: 1.03 ± 0.51/0.30 ± 0.18/31.8 ± 19.2% AUC _ELF_/AUC _plasma_ penetration ratio (mean ± SD) was 0.33 ± 0.48 PTA in plasma fT > MIC of 60 to 70% was < 90% for MICs of > 0.03 mg/L PTA in ELF T > MIC of 60% and 70% were 90.26% and 86.65%, respectively, at a MIC of 0.0125 mg/L and were significantly less for higher MICs	Cefditoren penetrates reasonably well into the ELF, as defined by the mean AUC_ELF_/AUC_plasma_ penetration ratio The overall probability of target attainment (T > MIC > 50%) in plasma and ELF, however, was suboptimal (< 90%)

*AC* alveolar cells, *AUC* area under the curve, *BAL* bronchoalveolar lavage, *CAP* community-acquired pneumonia, *C*_*max*_ maximal concentration, *C*_*12*_ concentration at 12 h, *C*_*min*_ trough concentration, *CFU* colony forming unit, *ELF* epithelial lining fluid, *ICU* intensive care unit, *LT* lung tissue, *MIC* minimal inhibitory concentration, *MIC90* minimal inhibitory concentration for 90% of isolates, *MRSA* methicillin-resistant *Staphylococcus aureus*, *P/T* piperacillin/tazobactam, *PTA* probability of target attainment, *SD* standard deviation, *T*_*1/2*_ half-life, *T > MIC* percentage of time above minimal inhibitory concentration, *VAP* ventilator-associated pneumonia

## New beta-lactam drugs and beta-lactamase combinations

Of great concern is the worldwide increase in the number of infections caused by Gram-negative multidrug-resistant bacteria. Treatment choices for these infections have been limited, especially for infections caused by bacteria that produce carbapenemases and/or extended-spectrum beta-lactamases.

Ceftolozane–tazobactam and ceftazidime–avibactam are 2 beta-lactams/beta-lactamase combinations with anti-Gram-negative bacteria activity that were recently approved for the treatment of complicated intra-abdominal infections, complicated urinary tract infections, and nosocomial pneumonia.

Ceftolozane is an oxyimino-aminothiazolyl cephalosporin with a pyrazole substituent at the 3-position side chain instead of the lighter pyridium present in ceftazidime. This heavier side chain provides improved steric hindrance to prevent hydrolysis mediated through AmpC beta-lactamases.

Ceftolozane–tazobactam combines a novel cephalosporin with an established beta-lactam beta-lactamase inhibitor, whereas ceftazidime–avibactam couples a well-known cephalosporin with a novel non-beta-lactam beta-lactamase inhibitor.

Both tazobactam and avibactam target the active site of serine beta-lactamases. Tazobactam, a beta-lactam sulfone, binds irreversibly to the active site of beta-lactamases and avibactam is a diazabicyclooctane non-beta-lactam that binds covalently and reversibly to beta-lactamases. This reversibility is a unique feature that allows avibactam to undergo recyclization to inactivate another beta-lactamase. The crucial advantage of avibactam is its ability to inhibit extended spectrum beta lactamases, AmpC beta-lactamases (as expressed in *Pseudomonas aeruginosa* and *Enterobacteriaceae*), and class A carbapenemases of the *Klebsiella pneumoniae* carbapenemase (KPC and OXA-48) family.

The pharmacokinetic and safety profiles of this antibiotic have been established in healthy adults and subjects with various degrees of renal function [170, 171]. The currently approved dosages for adult patients with an estimated ClCr > 50 mL/minute are ceftolozane 1 g with tazobactam 500 mg every 8 h and ceftazidime 2 g with avibactam 500 mg every 8 h for complicated urinary tract infections and intra-abdominal infections [172] and ceftolozane 2 g with tazobactam 1 g every 8 h for nosocomial pneumonia [173].

However, data guiding its use in critically ill patients are currently sparse, being entirely derived from studies with very few patients and/or case reports.

Veillete et al. [174] presented PK data for ceftazidime–avibactam from two patients with bloodstream infections caused by carbapenemase (KPC)-producing *K. pneumoniae*; the patients had renal impairment and one of them was obese. In both patients half-lives were prolonged and Vd larger than predicted. They conclude that recommended doses and intervals may not be sufficient for obese patients with renal failure, especially for those infected with KPC-producing organisms [174].

Oliver et al. [175] evaluated the adequacy of extended-infusion ceftolozane–tazobactam to achieve target PK and PD goals in a critically ill patient with *Pseudomonas aeruginosa* pneumonia and septic shock on CVVH. A dosage of 1.5 g every 8 h (3-h infusion) was given. All estimated plasma-free drug concentrations achieved the PD goals and remained well above the isolated organism’s MIC of 1.5 μg/mL and above the susceptibility breakpoint of 4 μg/mL throughout the dosing interval, although the authors could not comment on drug concentrations at the site of infection. The authors conclude that, given the lowest estimated free-drug concentration was fivefold greater than the susceptibility breakpoint, the estimated half-life of 28 h and the low extraction ratio observed, a lower total daily dose might be utilized and an extended infusion time may not be necessary for patients on CVVH [175].

Bremmer et al. [176] performed a PK analysis of intravenous ceftolozane–tazobactam 3 g every 8 h in a critically ill patient with *P. aeruginosa* pneumonia on CVVHDF. They concluded that, compared with a patient with normal renal function, this patient had decreased ceftolozane clearance. A ceftolozane–tazobactam dosage of 1.5 g every 8 h should adequately achieve a desired drug concentration above the minimum inhibitory concentration of 8 μg/mL for the treatment of pneumonia [176].

Stokem et al. reported the successful treatment with ceftolozane–tazobactam 3 g every 12 h for a pulmonary exacerbation in a 35-year-old female post-lung transplant, with cystic fibrosis, malnutrition, chronic kidney disease, and multi-drug resistant *P. aeruginosa* infection. Optimal time above MIC (estimated 100% time above MIC of ceftolozane achieved against both isolates was 2 and 0.5 μg/mL) was likely attained at the dose and frequency provided in this case [177].

## Toxicity

Beta-lactams are generally considered to have a high safety window with relatively few adverse effects, even when high doses are used [15]. Neurotoxicity is the most reported serious adverse effect of beta-lactams. Benzylpenicillin, cefepime, ceftazidime, and imipenem are considered to be the high-risk beta-lactams for neurotoxicity. Renal impairment, excess doses and/or concentrations, age, and a prior history of neurological disorders are known to be predisposing factors [2, 178–184].

Other adverse effects are found in a few case reports: acute renal failure [185] and electrolyte disorders [186]; severe intravascular haemolysis [187, 188]; extreme thrombocytosis [189]; severe thrombocytopenia [190–193]; leukopenia [194]; delayed-type hypersensitivity [195]; anaphylactic shock [196]; and severe cutaneous reactions [197].

## Therapeutic dose monitoring

Several studies reported high PK variability of beta-lactams in sepsis/septic shock, both in different patients and in the same patient over time. In critically ill patients, hydrophilic and moderately lipophilic antimicrobials, being at higher risk of daily PK variations, should be more closely monitored and their dosages should be streamlined according to the underlying diseases in order to prevent under- or overexposure [2, 11].

Therapeutic drug monitoring (TDM) has been instituted for aminoglycosides and glycopeptides to reduce the rate of toxicity. However, because of the safety profile of beta-lactams, TDM was thought unnecessary for these drugs. In line with PK changes in critically ill patients, insufficient PD target attainment with beta-lactams has been reported in these patients, especially those with hypoalbuminemia, altered renal function, and low susceptibility bacterial strain infections [2, 35, 42, 198]. The challenges in achieving ‘optimal’ drug concentrations in the critically ill suggest beta-lactam TDM as a useful strategy to optimize drug exposure [199].

The TDM approach could be particularly useful in a certain group of critically ill patients in whom achieving target concentrations is more difficult, such as those with highly resistant bacterial strains, obese patients, immunocompromised patients, those undergoing renal-replacement therapies, and patients with augmented renal clearance [2, 198, 200, 201].

Though there are PK models to estimate antibiotic concentrations over a range of creatinine clearance (CrCl) and on renal replacement therapy [67, 202–205], the use of CrCl as a tool to optimize beta-lactam dosing may not be reliable; although CrCl was significantly correlated with concentrations and clearance of broad-spectrum beta-lactams, changes in CrCl and RRT parameters do not reliably predict variations in drug PK/PD. In this setting, routine TDM should be considered to adapt beta-lactam doses [206].

Daily TDM of beta-lactams with dose adaptation in critically ill patients improves PD target attainment [207, 208]. Case reports have shown that TDM improved clinical outcome [209], but the clinical efficacy of using drug levels to achieve adequate concentrations had never been properly evaluated [1, 35, 210, 211] and there are reports concerning cost-effectiveness [111].

Facing poor implementation in beta-lactam TDM, Delattre et al. [212] proposed a predictive PK performance between an aminoglycoside and a beta-lactam. Due to physicochemical and PK similarities between aminoglycosides and beta-lactams, optimization of the beta-lactam dosage could be reached without any beta-lactam measurements, using TDM-related data of an aminoglycoside. The study aimed to characterize the PK of four beta-lactams (piperacillin, ceftazidime, cefepime, and meropenem) at the first dose in 88 critically ill septic patients co-medicated with amikacin, and to confirm the predictive performance of amikacin data on these PK, on a larger patient cohort, using a nonlinear mixed-effects modeling approach. There was a significant relationship between the exposure to amikacin and to beta-lactams. The population model presented was able to guide dosage adjustments for piperacillin, ceftazidime, cefepime, and meropenem during the early phase of severe sepsis in critically ill patients, using renal biomarkers or TDM-related aminoglycoside data [212].

## Conclusions

The duration of infusion of beta-lactams has been shown to influence their fT > MIC and an improved PD profile of beta-lactams may be obtained by longer exposure with more frequent dosing, extended infusions, or continuous infusions. This is particularly relevant in the critically ill patient, as Vd and ClCr are often increased, namely in the early phase of systemic hyperinflammatory states, promoting the risk of antibiotic underdosing.

The use of extracorporeal support techniques, either for renal replacement or ECMO, may further contribute to this problem and consequently concentrations below those expected are often found for beta-lactams. Given the heterogeneity of extracorporeal support therapy modes, it is difficult to suggest a specific dosage, but we recommend not to reduce dosage since no drug accumulation was found in the available literature and to use continuous or prolonged infusions to achieve the adequate PD profiles necessary to successfully treat infections caused by less susceptible strains.

More studies are needed to define optimal dosing of new beta-lactams and new beta-lactam/beta-lactamase combinations, which are increasingly important to effectively treat multidrug-resistant bacterial strains, namely in patients on extracorporeal support therapy and with difficult-to-treat sites of infection.

Although, it is not currently a clinical routine in most hospitals and its clinical efficacy has not yet been properly evaluated, a beta-lactam TDM approach with daily dose adaptation, allowing personalized antibiotic dosing, should be particularly useful in critically ill patients in whom achieving target concentrations is more difficult, such as obese patients, the immunocompromised, patients with augmented renal clearance, those undergoing extracorporeal support therapy, or those infected with highly resistant bacterial strains. Studies comparing TDM- versus non-TDM-based beta-lactam regimens should be promoted.

However, infection usually occurs at extravascular sites and prediction of outcome based on antibiotic plasma concentrations may result in overestimation of antimicrobial activity at the site of infection. Very few studies have investigated PK/PD issues concerning special anatomic barriers like the brain and lung, but most suggest that standard ICU dosing for beta-lactams may be insufficient for low susceptibility/high MIC pathogens in these sites. Therefore, although no studies have assessed clinical outcome, we recommend using higher than standard dosing, preferably with continuous or prolonged infusions, when treating severe infections caused by less susceptible bacterial strains at these sites, as PD profiles may improve and toxicity does not seems to increase.

## Abbreviations

AKI: Aacute kidney injury
AUC: Aarea under the curve
C: Concentration
CFU: Colony-forming units
CI: Continuous infusion
Cl: Clearance
ClCr: Creatinine clearance
CNS: Central nervous system
CRRT: Continuous renal replacement therapy
CSF: Cerebral spinal fluid
CVVHD: Continuous venous-venous hemodialysis
CVVHDF: Continuous venous-venous hemodiafiltration
CVVHF: Continuous venous-venous hemofiltration
ECMO: Extracorporeal membrane oxygenation
EDD: Extended daily dialysis
ELF: Epithelial lining fluid
ICU: Intensive care unit
II: Intermittent infusion
KPC: Carbapenemase producing *Klebsiella*
MBC: Minimal bactericidal concentration
MIC: Minimum inhibitory concentration
PAE: Post-antibiotic effect
PD: Pharmacodynamics
PK: Pharmacokinetics
PTA: Probability of target attainment
RRT: Renal replacement therapy
SLED: Sustained low-efficiency dialysis
TDM: Therapeutic drug monitoring
VAP: Ventilator associated pneumonia
Vd: Volume of distribution
